# *Bacillus thuringiensis* Crystal Protein Cry6Aa Triggers *Caenorhabditis elegans* Necrosis Pathway Mediated by Aspartic Protease (ASP-1)

**DOI:** 10.1371/journal.ppat.1005389

**Published:** 2016-01-21

**Authors:** Fengjuan Zhang, Donghai Peng, Chunsheng Cheng, Wei Zhou, Shouyong Ju, Danfeng Wan, Ziquan Yu, Jianwei Shi, Yaoyao Deng, Fenshan Wang, Xiaobo Ye, Zhenfei Hu, Jian Lin, Lifang Ruan, Ming Sun

**Affiliations:** 1 State Key Laboratory of Agricultural Microbiology, College of Life Science and Technology, Huazhong Agricultural University, Wuhan, China; 2 Haikou Experimental Station, Chinese Academy of Tropical Agricultural Sciences, Haikou, China; Stanford University, UNITED STATES

## Abstract

Cell death plays an important role in host-pathogen interactions. Crystal proteins (toxins) are essential components of *Bacillus thuringiensis* (Bt) biological pesticides because of their specific toxicity against insects and nematodes. However, the mode of action by which crystal toxins to induce cell death is not completely understood. Here we show that crystal toxin triggers cell death by necrosis signaling pathway using crystal toxin Cry6Aa-*Caenorhabditis elegans* toxin-host interaction system, which involves an increase in concentrations of cytoplasmic calcium, lysosomal lyses, uptake of propidium iodide, and burst of death fluorescence. We find that a deficiency in the necrosis pathway confers tolerance to Cry6Aa toxin. Intriguingly, the necrosis pathway is specifically triggered by Cry6Aa, not by Cry5Ba, whose amino acid sequence is different from that of Cry6Aa. Furthermore, Cry6Aa-induced necrosis pathway requires aspartic protease (ASP-1). In addition, ASP-1 protects Cry6Aa from over-degradation in *C*. *elegans*. This is the first demonstration that deficiency in necrosis pathway confers tolerance to Bt crystal protein, and that Cry6A triggers necrosis represents a newly added necrosis paradigm in the *C*. *elegans*. Understanding this model could lead to new strategies for nematode control.

## Author Summary

Necrosis contributes to many devastating pathological conditions, such as neurodegenerative diseases and microbial pathogenesis. *Bacillus thuringiensis* crystal proteins are effective biopesticides. Our study reveals that *B*. *thuringiensis* Cry6Aa protein triggers the necrosis pathway using *Caenorhabditis elegans* as a model. We show that aspartic protease ASP-1 is required for Cry6Aa protein-induced necrosis, whereas intrinsic insults induce necrosis mediated by ASP-3 and ASP-4. Our findings contribute to the understanding of the mechanism of Bt crystal protein action and host-pathogen interactions. Because necrosis mechanisms are conserved from nematodes to humans, the fact that necrosis can be induced by Cry6Aa provides a model system for studying necrosis mechanisms in human diseases.

## Introduction

Cell death plays critical roles in development and in pathological conditions. Apoptosis and necrosis are the two major modes of cell death [1]. Apoptosis, the most well- known mode of the cell death, plays a significant role in development, tissue homeostasis, and host defense [2,3]. Unlike apoptosis, necrosis is characterized by loss of plasma membrane integrity [4,5]. Necrotic cell death can contribute to many pathological conditions, such as inflammation [6], human neurodegenerative and aging-associated diseases [5,7]. Moreover, Necrosis plays an important role in microbial pathogenesis. In some cases, necrosis plays a significant role in antiviral/antibacterial host defense [2,8]; in others, necrosis is utilized as pathogen survival strategy to aid its spread [2].

*Bacillus thuringiensis* (Bt) is one member of the *Bacillus cereus* group of bacteria [9]. An important characteristic of Bt strains is that they produce insecticidal crystal proteins (Cry) during the sporulation phase. These proteins are highly specific to their target insects and nematodes and are harmless to non-target animals and humans, thus, they represent a viable alternative for the control of pests in agriculture and of important disease vectors in human public health [10].

The classical pore-forming model remains the widely accepted mode of action of the three-domain crystal protein (3d-Cry). When susceptible larvae ingest the 3d-Cry protoxin, it is activated by gut proteases. Upon activated toxin binding to cadherin receptor, toxins forms oligomers. Subsequently, these oligomers bind to a second group of receptor proteins. Finally, they generate toxin pores in the cell membrane that leading to midgut cells lysis [10–13]. There is little information on the cellular mechanisms of the classical pore-forming model. An alternative signaling pathway model of the 3d-Cry action has also been reported. This model disregards pore formation and proposed that crystal toxins activate a Mg^2+^-dependent adenylyl cyclase (AC)/protein kinase A (PKA) signaling pathway by interacting with receptor [14,15]. Nevertheless, this signaling pathway has only been identified in one insect cell line. There is no published data showing a signaling pathway involved in whole larvae death and the mode of action by which crystal toxins to induce cell death is not completely understood.

Crystal proteins have also been shown to intoxicate nematode parasites of animals [16] and plants [17–19]. Nematicidal activity has been found in several families of Bt crystal proteins, including Cry5, Cry6, Cry12, Cry13, Cry14, Cry21, and Cry55 [17]. Cry5Ba and Cry6Aa represent two distinct families. The structure of Cry5Ba is similar to that of insecticidal 3d-Cry, and shows the conserved three-domain (3-d) architecture responsible for pore formation in insecticidal crystal proteins [20]. However, Cry6Aa does not show the typical 3-d architecture. In contrast to the insect model, the mode of action of nematicidal crystal toxins has been investigated only in *C*. *elegans* using the Cry5Ba [21]. As previously shown, the receptors for crystal protein Cry5Ba in *C*. *elegans* are invertebrate-specific glycolipids [21]. The Cry5Ba-resistant glycolipid mutants are sensitive to Cry6Aa [22]. The above information implies that the Cry6Aa may utilize a different toxicity pathway from Cry5Ba.

In *C*. *elegans*, necrotic cell death can be induced by extreme environmental stimuli or intrinsic insults, including hypoxia [23], ionic imbalance, heat stroke, bacterial infection, and hypoosmotic shock [24]. In this study, we found that necrotic cell death can also be induced by *B*. *thuringiensis* crystal protein Cry6Aa. This pathway induced by Cry6Aa is mediated by aspartic protease (ASP-1). Additionally, ASP-1 protects the crystal protein Cry6Aa from over-degradation by *C*. *elegans*.

## Results

### ASP-1 is required for Cry6Aa-mediated toxicity

Proteomic approaches have previously been used to identify *B*. *thuringiensis* toxin binding proteins [25] and toxin receptors [26] and to understand insect resistance to *B*. *thuringiensis* [27]. Cry6Aa binding proteins in *C*. *elegans* were identified using 2-DE proteomics and ligand blotting. Separation of the *C*. *elegans* proteins by isoelectric focusing with a pH 4–7 IPG strip (18 cm) revealed that most proteins detected in 2-DE separations were smaller than 175 kDa and fell in the pH range of 4–6 (S1A Fig). Two-dimensional gel blots were probed with biotin-labeled Cry6Aa to identify *C*. *elegans* proteins that bound to Cry6Aa (S1B Fig). Fourteen silver-stained protein spots were detected (S1A Fig) and selected for identification. Four of these proteins were identified by mass spectroscopy and peptide mass fingerprinting (PMF) (S1 Table), whereas the other spots were not identified. Spot 1 matched aspartic protease (ASP-1) from *C*. *elegans*, and Spots 2, 3 and 4 matched an ATP synthase subunit family member (ATP-2), actin and F58E2.4, respectively.

**Fig 1:**
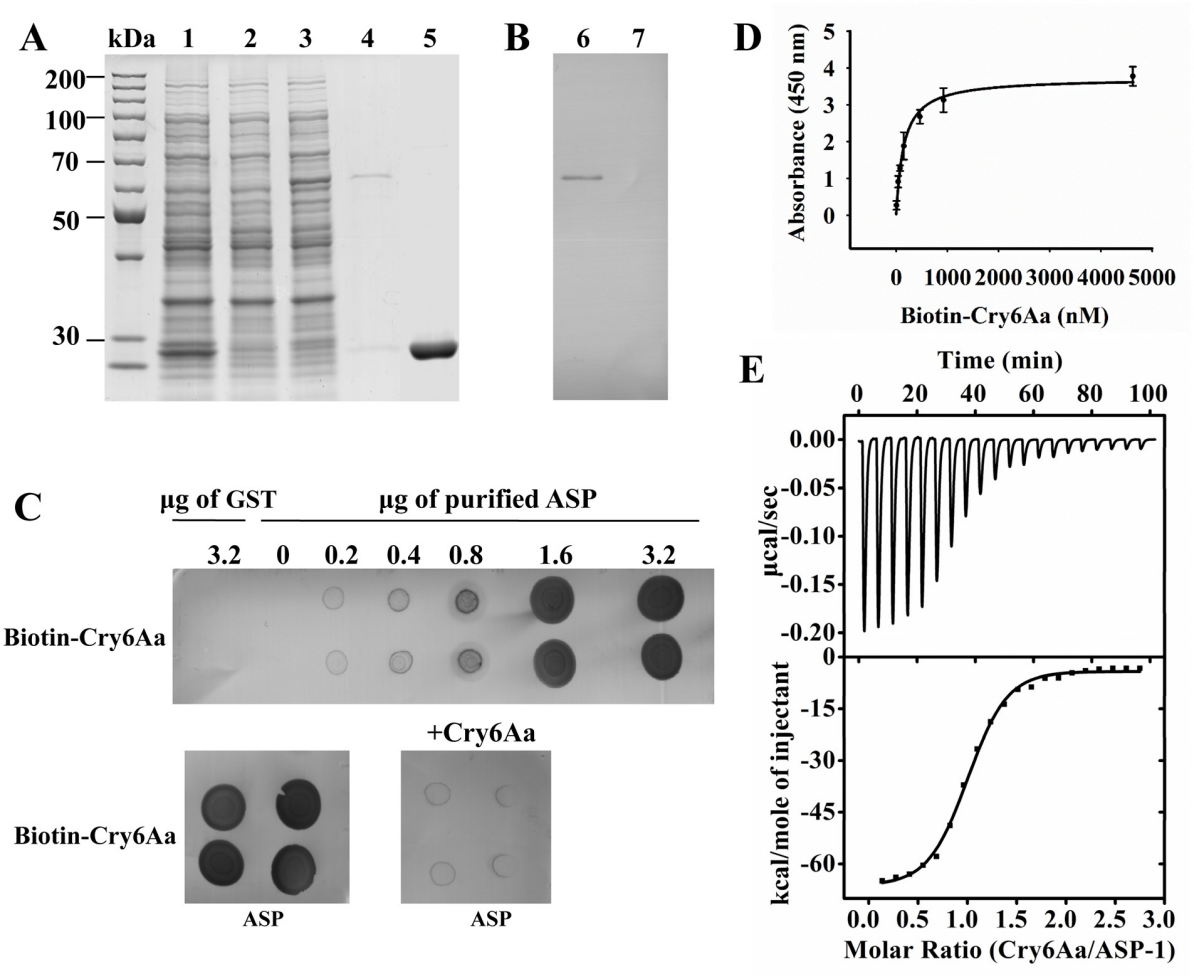
The specific interactions between Cry6Aa and ASP-1. (A) The expression and purification of ASP1-GST fusion protein in *E*. *coli*. Lane 1, *E*. *coli* BL21 containing pGEX-6p-1 vector with 0.1 mM IPTG induction under 16°C overnight, cell pellet; lane 2, *E*. *coli* BL21 containing pASP1-GSTvector without IPTG induction, cell pellet. lane 3, *E*. *coli* BL21 containing pASP1-GST vector with 0.1 mM IPTG induction under 16°C overnight, cell pellet. lane 4, purified ASP1-GST fusion protein eluted from the glutathione Sepharose bulk; lane 5, purified GST tag eluted from the glutathione Sepharose bulk. (B) The binding of Cry6Aa to ASP1-GST fusion proteins by ligand blotting. The purified ASP1-GST fusion proteins (lane 6) or GST (lane 7) were separated by SDS-PAGE gels, and were transferred to a PVDF membrane. Filters were blocked overnight, and then probed with biotinylated Cry6Aa, Unbound toxin was removed by washing. The bound protein was detected streptavidin-horseradish peroxidase (HRP) conjugate. And finally the membrane was visualized. GST was the control. (C) ASP-1 proteins were dotted on a NC membrane directly and were probed with biotin labeled Cry6Aa or with biotin labeled Cry6Aa plus unlabeled Cry6Aa (1000-fold). GST was the control. (D) Binding affinity of Cry6Aa to ASP-1 was determined by ELISA. Ninety-six-well microtiter plates coated with ASP-1 were incubated with increasing concentrations of biotinylated Cry6Aa alone or with 1000-fold molar excess of unlabeled Cry6Aa to determine specific binding. Each point represents mean amounts of protein specifically bound. Specific binding was determined by subtracting nonspecific binding (with 1000-fold molar excess of unlabeled Cry6Aa) from total binding (without excess unlabeled Cry6Aa). (E) Isothermal titration calorimetric analysis of Cry6Aa binding to ASP-1. Titration of ASP-1 (2.20 μM) with 21.16 μM Cry6Aa. The top panel show the raw data of the heat released, and the bottom panel show the binding isotherm fitted using nonlinear binding models. Data were analyzed in Origin 8.6 software after subtracting the heat released from titrating Cry6Aa alone into buffer. One of three representative experiments is shown.

ASP-1 is mainly distributed in the intestinal cells of *C*. *elegans* [28]. The toxicity of crystal protein Cry6Aa toward *C*. *elegans* is believed to involve intestinal damage [29]. Therefore, ASP-1 is likely to be involved in Cry6Aa toxicity against *C*. *elegans*. To further characterize the interaction between ASP-1 and Cry6Aa, a ASP1-GST fusion protein was over-expressed in *Escherichia coli* (Fig 1A, lane 3) and was purified by affinity chromatography with glutathione Sepharose 4B (Fig 1A, lane 4). Ligand blot experiments showed that crystal protein Cry6Aa can bind to purified ASP1-GST fusion protein (Fig 1B, lane 6). In the control experiment, Cry6Aa did not bind to purified GST (Fig 1B, lane 7).

To facilitate the measurement of Cry6Aa toxin binding under non-denaturing conditions, purified ASP1-GST was dotted in increasing amounts on a membrane filter, and the filter was probed with biotin-labeled Cry6Aa. GST was a negative control, dot blot showed 3.2 μg GST did not bind to Cry6Aa (Fig 1C). As the amount of dotted ASP1-GST protein increased, more biotin-labeled Cry6Aa was bound, and excess unlabeled Cry6Aa (1000-fold) competed for biotin-labeled Cry6Aa binding, which indicated that the binding between Cry6Aa and ASP1-GST was specific (Fig 1C). To measure the binding affinity of Cry6Aa to ASP1-GST, a competitive ELISA was performed. The dissociation constant (Kd) for ASP1-GST binding to Cry6Aa was 145.7 nM (126.8–164.7 nM) (Fig 1D). To assess whether ASP-1 and Cry6Aa interact directly, we determined the binding affinity between ASP-1 and Cry6Aa by isothermal titration calorimetry (Fig 1E). The estimated Kd was 126.4 nM (99.0–153.8 nM) (Fig 1E).

**Fig 2:**
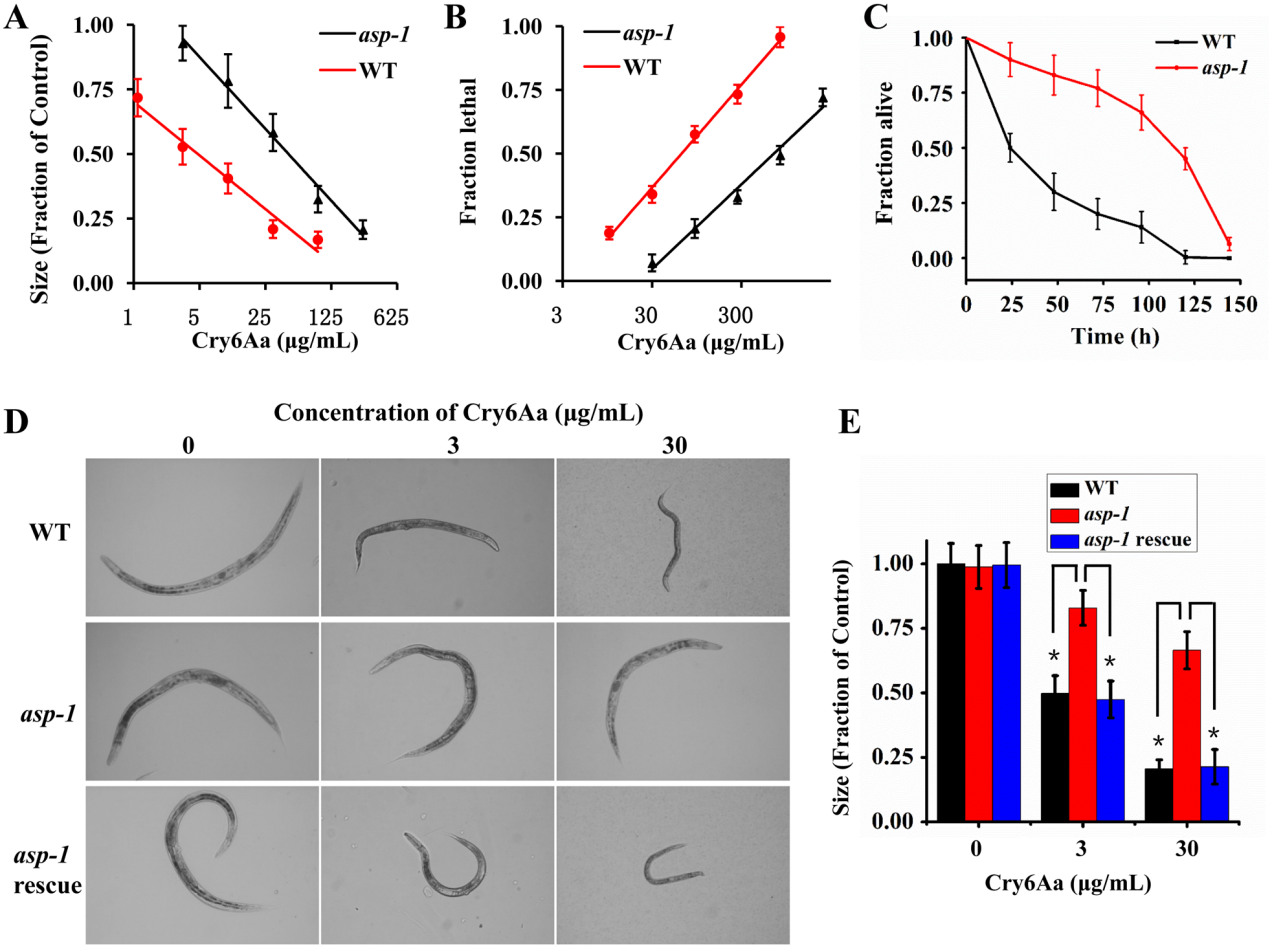
The susceptibility of mutant *asp-1(tm666)* to Cry6Aa. (A) Cry6Aa quantitative growth assay with wild type N2 and mutant *asp-1(tm666)*. (B) Cry6Aa lethality assay with N2 and *asp-1(tm666)*. The strains (as shown) were exposed to five doses of Cry6Aa. (C) Survival of wild type N2 and mutant *asp-1(tm666)* exposed to Cry6Aa. (D) Growth assay of different concentrations of Cry6Aa against L1 larvae of N2 (top panels), *asp-1(tm666)* (middle panels), and *asp-1* (*tm666*) rescued with the whole *asp-1* gene (bottom panels) observed under a light microscope. (E) Cry6Aa quantitative growth assay with N2, *asp-1(tm666)* and *asp-1(tm666)* transformed with the whole *asp-1* gene. Data were showed as mean ± SD (n = 3).

To test the role of Asp-1 *in vivo*, we compared the difference in larval growth inhibitory activities (GI) and mortality after treatment with Cry6Aa between wild type *C*. *elegans* N2 and mutant *asp-1* (*tm666*). For the quantitative growth test, the GI_50_ values for N2 and *asp-1* (*tm666*) were 4.9 ± 0.6 μg/mL and 45.1 ± 3.7 μg/mL, respectively (S2 Table and Fig 2A). Their LC_50_ values were 63.7 ± 6.8 μg/mL and 715.6 ± 69.3 μg/mL, respectively (S3 Table and Fig 2B). Lifespan measurements were performed in N2 and *asp-1(tm666)* upon exposure to Cry6Aa. As expected, the fraction of *asp-1(tm666)* alive was significantly higher than that of N2 (Fig 2C). The effects of different concentrations of Cry6Aa on the growth of L1 larvae of *C*. *elegans* N2 (top panels) and mutant *asp-1(tm666)* (middle panels) are summarized in Fig 2D. L1 larvae of N2 (Fig 2D, top panels) were not able to progress to adulthood after exposure to 30 μg/mL Cry6Aa. However, the L1 larvae of mutant *asp-1(tm666)* (Fig 2D, middle panels) were able to progress to adulthood after the same exposure. These experiments indicated that the mutation of *asp-1* reduces the nematode’s susceptibility to Cry6Aa.

When we transformed *asp-1* (*tm666*) with the whole *asp-1* gene, the rescued L1 larvae of *asp-1* (*tm666*) were unable to progress to adulthood after exposure to 30 μg/mL Cry6Aa (Fig 2D, bottom panels, and Fig 2E), which indicates that the whole *asp-1* gene was sufficient to restore susceptibility to Cry6Aa in the *asp-1* (*tm666*) mutant background. Taken together, these results indicate that ASP-1 is required for Cry6Aa-induced *C*. *elegans* death.

#### ASP-1 mediated protection of Cry6Aa in *C*. *elegans*

Bt protoxins are often activated to smaller active toxin, and insect midgut proteases are essential for the activation of Bt protoxins [30]. To monitor the size of Cry6Aa that is found inside the nematode, N2 and *asp-1(tm666)* were fed purified Cry6Aa proteins. Total proteins were then extracted from crystal protein treated nematodes, separated by SDS-PAGE, and subjected to Western blot analysis using an anti-Cry6Aa antibody. Only the fragment A2 (S2 Fig, arrow in black) is present in N2 background. However, in the *asp-1(tm666)* background, both fragment A2 (S2 Fig, black arrow) and fragment A3 (S2 Fig, blue arrow) coexist.

When Cry6Aa protoxin (S3A Fig, band A1, arrow in red) was digested by crude protein extracts from *C*. *elegans* N2 and *asp-1* (*tm666*), only the fragment A2 (S3A Fig, arrow in black) is present in N2 background. However, in the *asp-1(tm666)* background, both fragment A2 (S3A Fig, black arrow) and fragment A3 (S3A Fig, blue arrow) coexist. A bioassay showed that the activities of the *asp-1 (tm666)* digested fragments were reduced compared with that of the full-length Cry6Aa toxin and the N2 digested fragments (S3B Fig). Therefore, fragment A2 (S3A Fig, black arrow) corresponds to the active toxin, and the over-degradation fragment A3 (S3A Fig, blue arrow) corresponds to the inactive toxin, which suggests that one of the roles of ASP-1 is to protect Cry6Aa against gut proteases.

To further demonstrate the protective effect of ASP-1, we pre-incubated Cry6Aa with ASP-1 and then exposed it to the extract from *asp-1* mutant worms and found that the misprocessed fragment disappeared (S3A Fig). This protective process restored the toxicity of the pre-incubated Cry6Aa to N2 (S3B Fig). These results provide additional support for the idea that ASP-1 mediates the protection and stabilization of Cry6Aa.

#### Cry6Aa triggers the necrosis pathway in *C*. *elegans*

**Fig 3:**
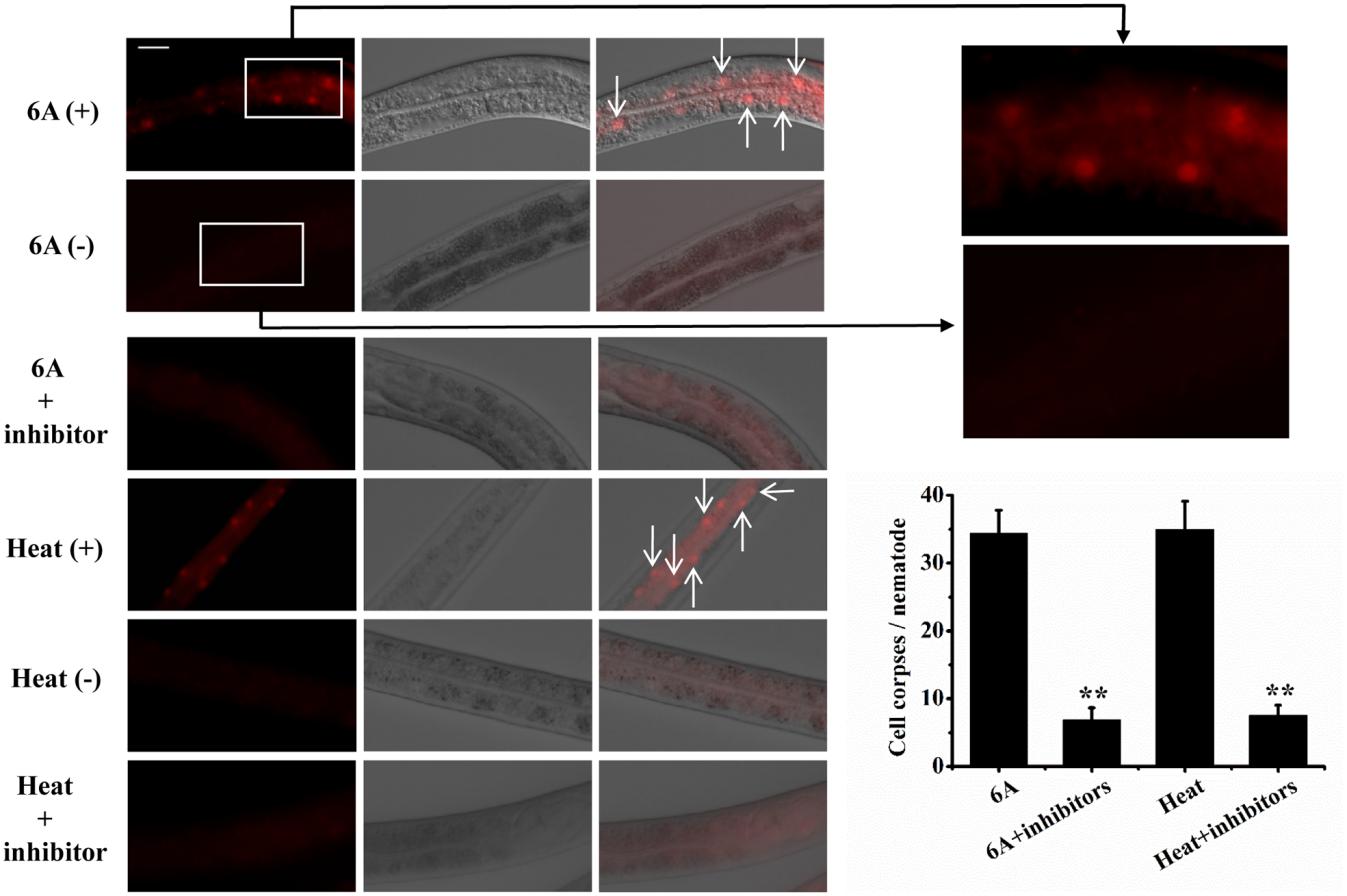
Intestinal cell plasma membrane integrity was lost in wild type nematode N2 after exposure to Cry6Aa or heat stroke. Heat stroke was a positive control. Fluorescence microscopy was used to monitor propidium iodide uptake. Arrows indicate intestinal cells stained with propidium iodide due to loss of membrane integrity. Two of the fluorescent images were magnified (boxed inset). The numbers of cell corpses per nematode were counted (Right). These results are the mean ± SD of three independent experiments. Double asterisks indicate p < 0.01. The bar denotes 20 μm.

Apoptosis and necrosis are the two major modes of cell death [1]. Propidium iodide (PI), which is a nucleic acid stain, acts as a marker for plasma membrane integrity, as it is only able to permeate cells when membrane integrity is disrupted, such as cells occur in necrosis [31,32]. Thus, PI can be used to distinguish between apoptotic cells and necrotic cells [31]. It is reported that propidium iodide is used as a marker of necrotic cell death [33,34]. When nematodes were exposed to Cry6Aa, intestinal cells of wild type nematode lost membrane integrity as shown by uptake of propidium iodide (Fig 3). However, the uptake of propidium iodide was significantly suppressed in nematode treated with necrosis inhibitors (Fig 3) or in some necrosis mutants (S4 and S5 Figs). Thus, we hypothesized that Cry6Aa might trigger necrotic cell death in the nematode.

It is reported that heat stroke triggered extensive necrotic cell death in *C*. *elegans* [35,36]. Therefore, heat stroke is used as a positive control in this study. As expected, when nematodes were exposed to heat stroke, intestinal cells of nematode lost membrane integrity as shown by uptake of propidium iodide (Fig 3). However, the uptake of propidium iodide was significantly suppressed in nematode treated with necrosis inhibitors (Fig 3).

**Fig 4:**
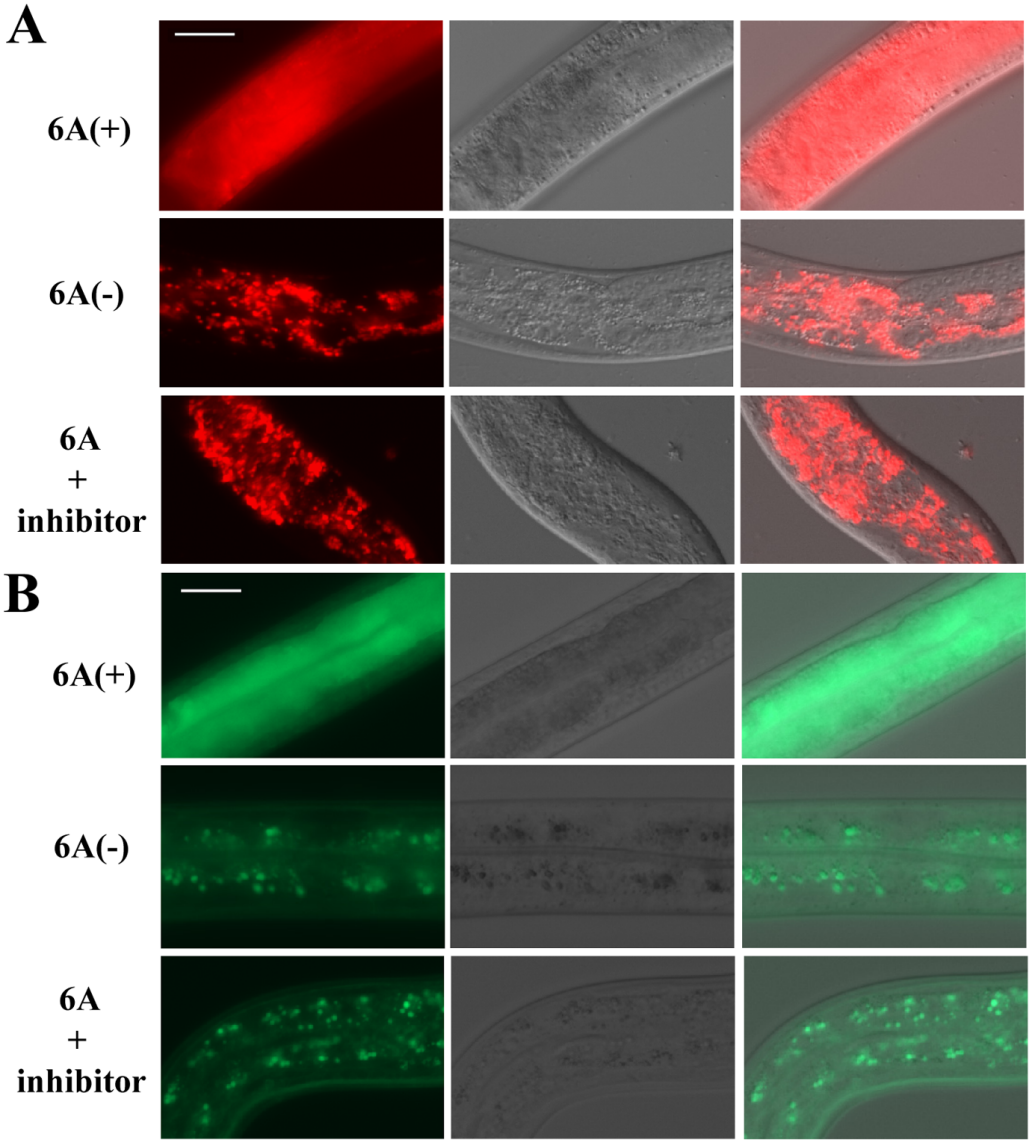
Cry6Aa-induced intestinal cell lysosomal rupture in *C*. *elegans*. (A) DIC and fluorescence microscopy of nematodes labeled with the intestinal lysosomal marker Lysotracker. (B) DIC and fluorescence microscopy of nematode RT258 (LMP-1::GFP strain). The intestinal lysosomal marker LMP-1::GFP was used to examine lysosomal integrity. The controls without Cry6A show significant staining, indicating that fluorescent gut lysosomes remained intact. In the Cry6A treated nematode, the labeled lysosome was found to be diffused (Fig 4A), indicating that Cry6Aa induces intestinal cell lysosomal rupture.

**Fig 5:**
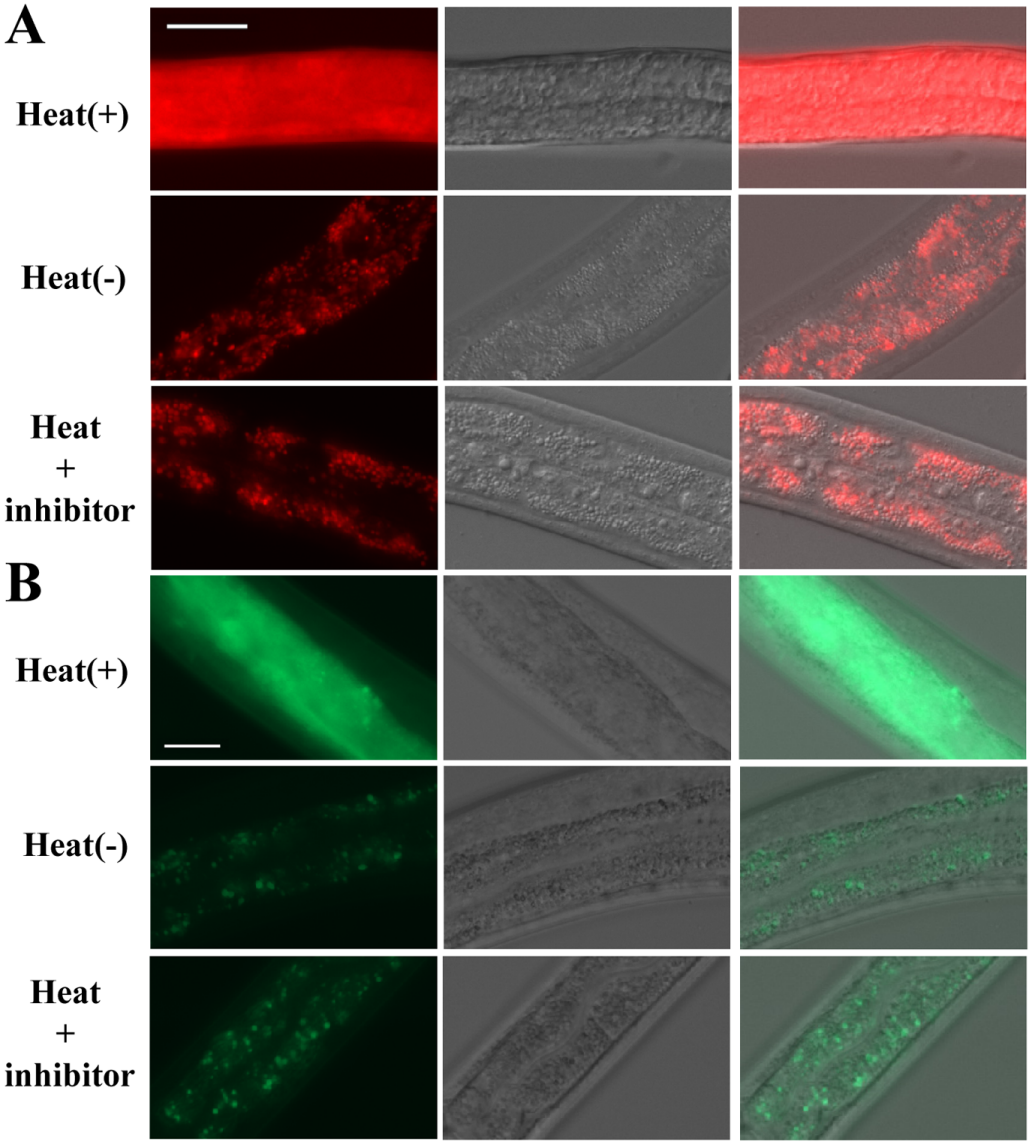
Heat stroke induced intestinal cell lysosomal rupture in *C*. *elegans*. (A) DIC and fluorescence microscopy of nematodes labeled with the intestinal lysosomal marker Lysotracker. (B) DIC and fluorescence microscopy of nematode RT258 (LMP-1::GFP strain). The intestinal lysosomal marker LMP-1::GFP was used to examine lysosomal integrity. In the nematode without heat stroke, Fluorescent gut lysosomes remained intact. In the heat stroke treated nematode, the loss of lysosomal membrane integrity was accompanied by diffused fluorescence.

Necrosis is characterized by the rupture of lysosomes [37,38]. We examined whether Cry6Aa induces intestinal cell lysosomal rupture. First, *in vitro*, the intestinal lysosomal marker lysotracker was used to examine the lysosomal integrity during Cry6Aa-induced death. As expected, killing with Cry6Aa (Fig 4A) or heat stroke (Fig 5A) resulted in a burst of red fluorescence, indicating gut granule lysis. Second, *in vivo*, a *C*. *elegans* transgenic (pwIs50) strain expressing the intestinal lysosomal marker LMP-1::GFP [39,40] was used to examine lysosome integrity. GFP-labeled lysosome was found to be diffused upon treatment with Cry6Aa (Fig 4B) or heat stroke (Fig 5B). However, the gut granule lysis was significantly suppressed in nematode treated with a necrosis inhibitor (Figs 4 and 5). These *in vivo* and *in vitro* experiments indicated that Cry6Aa induces intestinal cell lysosomal rupture.

**Fig 6:**
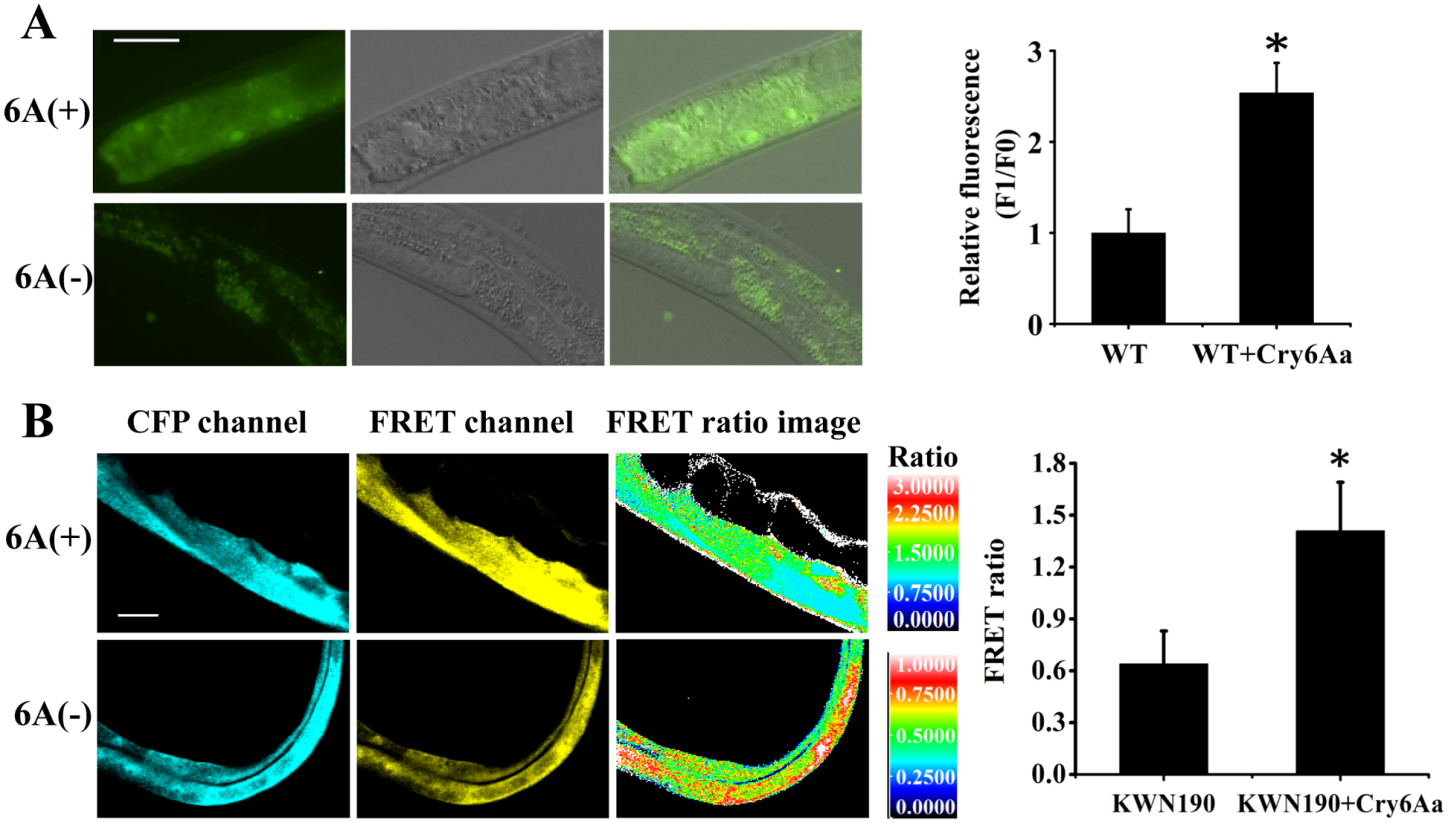
Cry6Aa-induced an increase in cytoplasm calcium concentration in *C*. *elegans*. (A) The rise in [Ca^2+^]_i_ in the intestine of wild type nematode N2 induced by 63 μg/mL Cry6Aa for 6 days. Fluorescence microscopy was used to monitor calcium concentration by measuring cytoplasmic fluorescence using the calcium indicator Fluo-4 AM. The right part shows the quantification of the fluorescence levels. These results are the mean ± SD of three independent experiments. A single asterisk indicates p < 0.05. The bar denotes 20 μm. (B) Representative CFP, FRET and Ratio images in transgenic nematode KWN190 upon exposure to 63 μg/mL Cry6Aa for 6 days. Calcium levels were visualized using the calcium indicator d3cpv expressed from the intestine-limited promoter in KWN190. *Right* shows that the FRET ratio increased upon exposure to Cry6Aa. The image is representative of three independent experiments. A single asterisk indicates p < 0.05. The bar denotes 20 μm.

An increase in the concentration of cytoplasmic calcium ([Ca^2+^]_i_) is required for activation of necrosis [41]. We examined whether Cry6Aa affects concentration of cytoplasmic calcium. First, *in vitro*, changes in [Ca^2+^]_i_ can be assessed by measuring cytoplasmic fluorescence using the calcium indicator Fluo-4 AM [36]. As expected, killing with Cry6Aa resulted in an increase in fluorescence of 1.5-fold (Fig 6A), which indicated an increase in [Ca^2+^]_i_. Second, *in vivo*, a *C*. *elegans* strain carrying an integrated transgene (rnyEx109) that expresses the calcium indicator d3cpv under the control of the intestine-limited promoter Pnhx-2 was used to monitor intracellular Ca^2+^ concentrations by fluorescence-resonance energy transfer (FRET) assay [42,43]. In this study, the relative cytosolic Ca^2+^ concentration is showed as the FRET ratio (defined as the fluorescence intensity in the FRET channel divided by the intensity in the CFP channel) [41,42]. The FRET ratio of controls without Cry6A was 0.64, indicating low levels of calcium. The FRET ratio with Cry6A was 1.41, indicating high levels of calcium. We observed a 120% increase in the FRET ratio upon exposure to Cry6Aa (Fig 6B), which indicated an increase in [Ca^2+^]_i_. These *in vivo* and *in vitro* experiments indicated that Cry6Aa induced an increase in cytoplasm calcium concentration.

At the organismal level, it is reported that necrotic death is accompanied by a burst of intense death fluorescence (DF) after the worms respond to the necrosis inducing stimuli [43]. As expected, both of heat stroke (S6 Fig) and Cry6Aa (S7A Fig) led to a burst of death fluorescence.

Taken together, the above results support the idea that Cry6Aa triggers the necrosis pathway in *C*. *elegans*.

### A deficiency in the necrosis pathway confers tolerance to Cry6Aa

Necrosis in *C*. *elegans* requires an increase in intracellular Ca^2+^ levels mediated by the inositol triphosphate receptor ion channel (ITR-1), the calcium-dependent cysteine protease mediated by calpain TRA-3, lysosomal lysis for cytosolic acidification mediated by the vacuolar proton translocating ATPase (VHA-12), and the destructive release of lysosomal killer cathepsin proteases mediated by ASP-3 [44] or ASP-4 [43,44].

**Fig 7:**
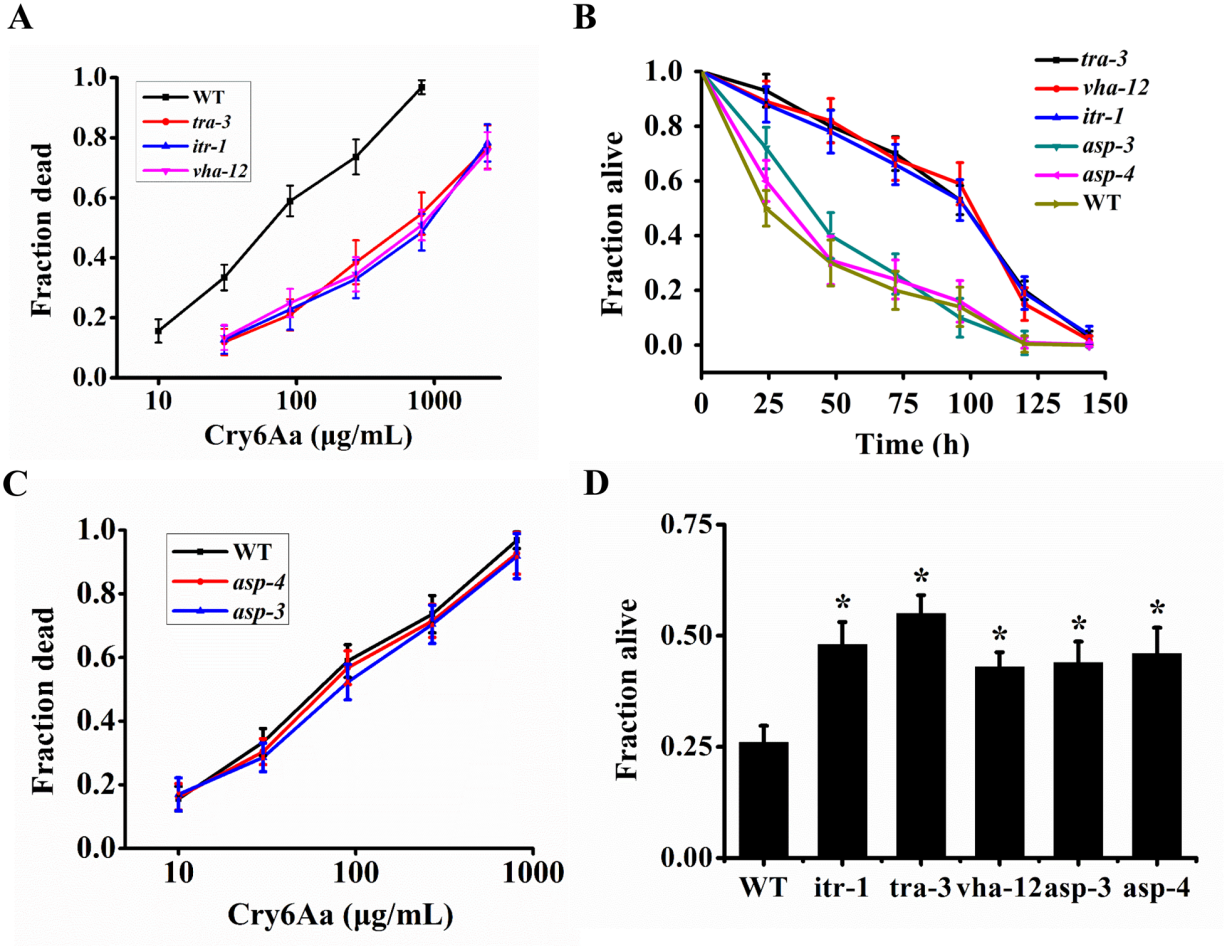
The susceptibility of necrosis mutants to Cry6Aa. (A) Cry6Aa lethality assay with *itr-1(sa73)*, *tra-3(e1107)*, and *vha-12(ok821)* necrosis mutants. The strains (as shown) were exposed to five doses of Cry6Aa. (B) Survival of *itr-1(sa73)*, *tra-3(e1107)*, *vha-12(ok821)*, *asp-3(tm4559)*, and *asp-4(ok2693)* necrosis mutants exposed to Cry6Aa. Mutations in *itr-1(sa73)*, *tra-3(e1107)*, and *vha-12(ok821)* reduced the sensitivity of nematodes to Cry6Aa. (C) Cry6Aa lethality assay with *asp-3(tm4559)* and *asp-4(ok2693)*. The strains (as shown) were exposed to five doses of Cry6Aa. (D) Survival of *itr-1(sa73)*, *tra-3(e1107)*, *vha-12(ok821)*, *asp-3(tm4559)*, and *asp-4(ok2693)* necrosis mutants exposed to heat stroke. Mutations in *itr-1(sa73)*, *tra-3(e1107)*, and *vha-12(ok821)* reduced the sensitivity of nematodes to heat stroke. A single asterisk indicates p < 0.05. Data were showed as mean ± SD (n = 3).

To examine whether necrosis is implicated in host defenses or for disease susceptibility during Cry6Aa targeting nematode, we compared the difference in mortality after treatment with Cry6Aa between wild type *C*. *elegans* N2 and necrosis mutants. Relative to the wild type, mutations in *itr-1(sa73)*, *tra-3(e1107)*, and *vha-12(ok821)* reduced the sensitivity of nematodes to Cry6Aa since their LC_50_ values were 614.9 ± 70.6 μg/mL, 539.8 ± 81.4 and 594.2 ± 68.5 μg/mL, respectively (S3 Table and Fig 7A). Lifespan measurements were performed in N2 and these mutations upon exposure to Cry6Aa. As expected, the fraction of *itr-1(sa73)*, *tra-3(e1107)*, and *vha-12(ok821)* alive was significantly higher than that of N2 (Fig 7B). These experiments indicated that these mutations are tolerant to the crystal protein Cry6Aa. However, neither the necrosis mutant *asp-3* (*tm4559*) nor *asp-4* (*ok2693*) significantly suppressed the induction of nematode death by Cry6Aa (S3 Table and Fig 7C). Heat stroke was a positive control, as expected, relative to the wild type, mutations in *itr-1(sa73)*, *tra-3(e1107)*, *vha-12(ok821)*, *asp-3* (*tm4559*) and *asp-4* (*ok2693*) reduced the sensitivity of nematodes to heat stroke (Fig 7D).

Taken together, these results indicate that a deficiency in the necrosis pathway confers tolerance to Cry6Aa.

#### ASP-1 is required for Cry6Aa-induced *C*. *elegans* necrosis

**Fig 8:**
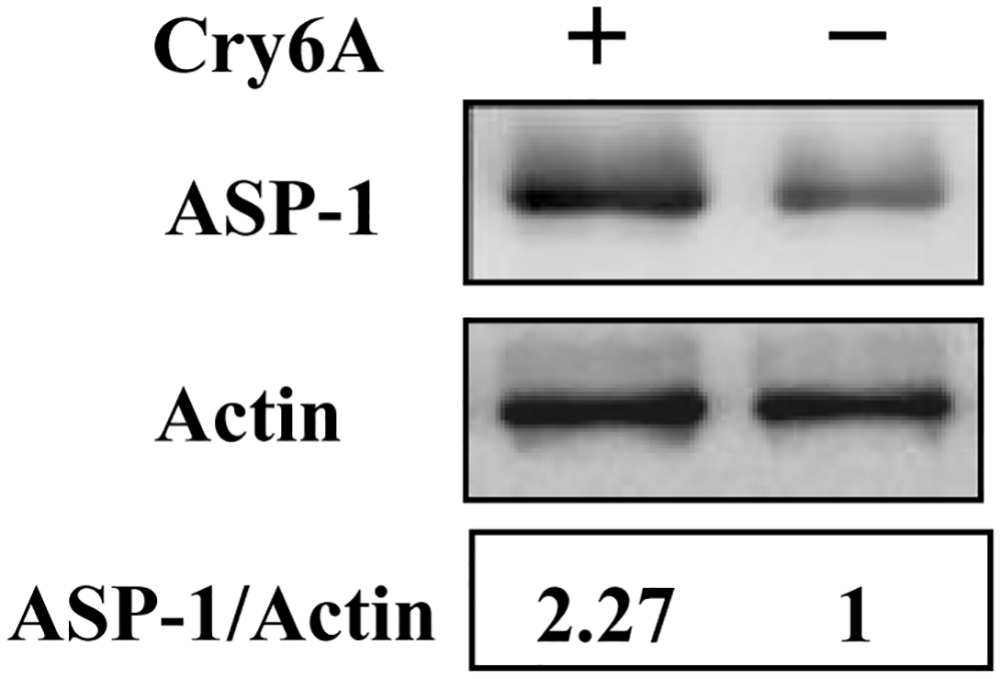
The protein expression of *asp-1* was up regulated in *C*. *elegans* upon exposure to Cry6Aa. Western blot shows the increased ASP-1 protein levels in *C*. *elegans* upon exposure to Cry6Aa. The blot is one of the three independent experiments.

It has been reported that necrosis requires the killer cathepsin lysosomal proteases to dismantle the cell [44]. ASP-1 is a typical cathepsin lysosomal protease [44], and the above results confirmed that *asp-1* is required for Cry6Aa-mediated toxicity (Fig 2). Western blotting showed a 127% increase in the ratio of ASP-1/Actin upon exposure to Cry6Aa, which indicated that the protein expression of *asp-1* was up regulated in *C*. *elegans* upon exposure to Cry6Aa (Fig 8). To determine whether *asp-1* is required for Cry6Aa-induced *C*. *elegans* necrosis, propidium iodide staining was performed in Cry6Aa treatment of the mutant *asp-1(tm666)*. As expected, the uptake of propidium iodide was significantly suppressed in *asp-1(tm666)* upon exposure to Cry6Aa (S7B Fig). In addition, *asp-1(tm666)* was able to reduce death fluorescence induced by Cry6Aa (S7A Fig). Examination of the intestinal lysosomal marker lysotracker showed that the burst of red fluorescence was significantly suppressed in *asp-1(tm666)* upon exposure to Cry6Aa (S7C Fig), indicating that *asp-1* is required for Cry6Aa-induced intestinal lysosomal rupture.

Together, these results suggest that the Cry6Aa-induced necrosis pathway is mediated by ASP-1.

#### The necrosis pathway is specifically induced by Cry6Aa, not by Cry5Ba

**Fig 9:**
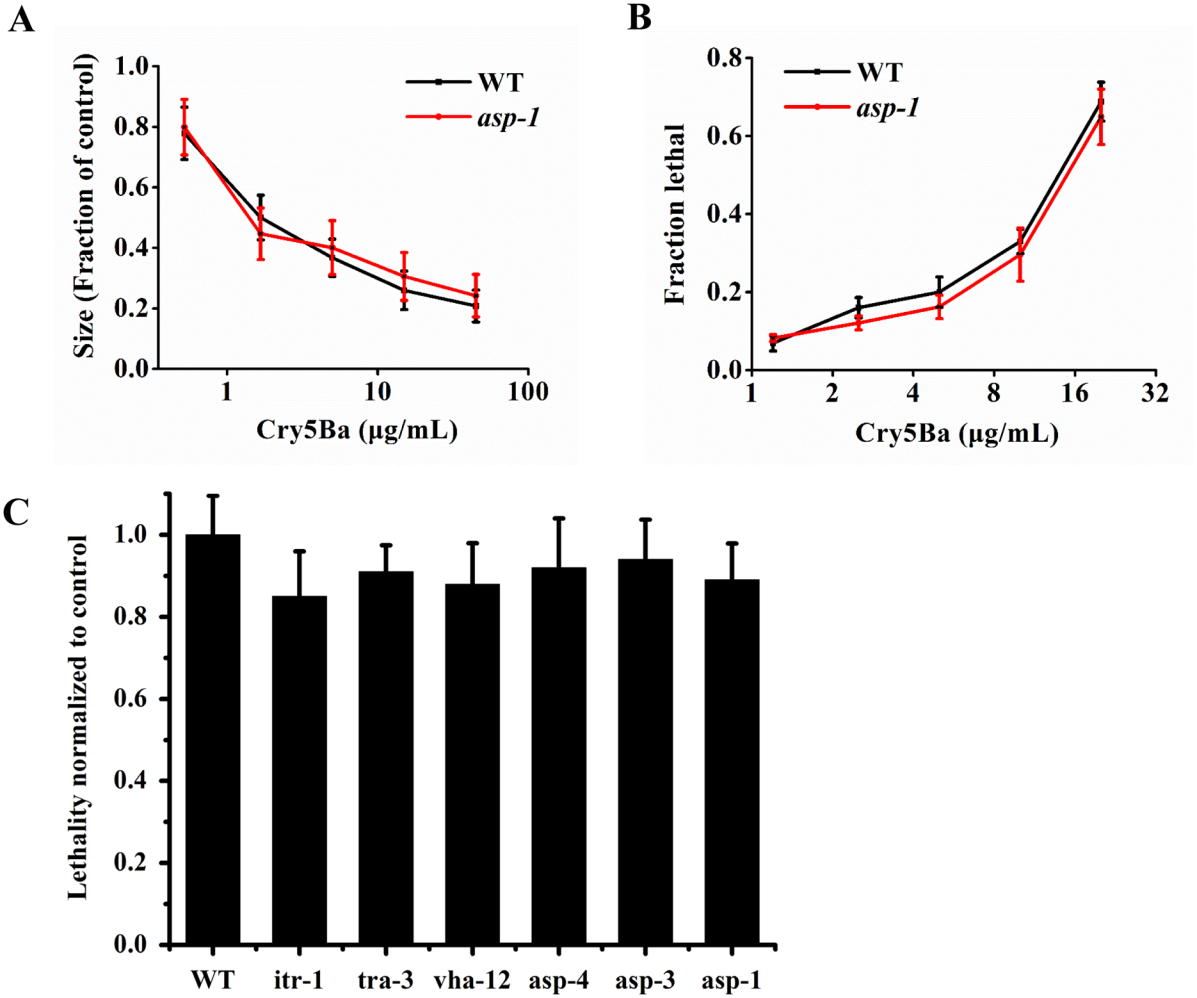
The susceptibility of necrosis mutants to Cry5Ba. (A) Cry5Ba quantitative growth assay with wild type N2 and mutant *asp-1(tm666)*. (B) Cry5Ba lethality assay with N2 and *asp-1(tm666)*. The strains (as shown) were exposed to five doses of Cry6Aa. (C) Lethality of necrosis mutants exposed to Cry5Ba. Data were showed as mean ± SD (n = 3).

To determine whether the mutant *asp-1(tm666)* was specifically tolerant to Cry6Aa or to nematicidal crystal proteins in general, we tested *asp-1(tm666)* for tolerance to another nematicidal crystal protein, Cry5Ba. Both mutant *asp-1(tm666)* and the wild-type N2 were sensitive to Cry5Ba at the same level (Fig 9A and 9B). In addition, none of the necrosis mutants suppressed Cry5Ba-induced death in *C*. *elegans* (Fig 9C).

**Fig 10:**
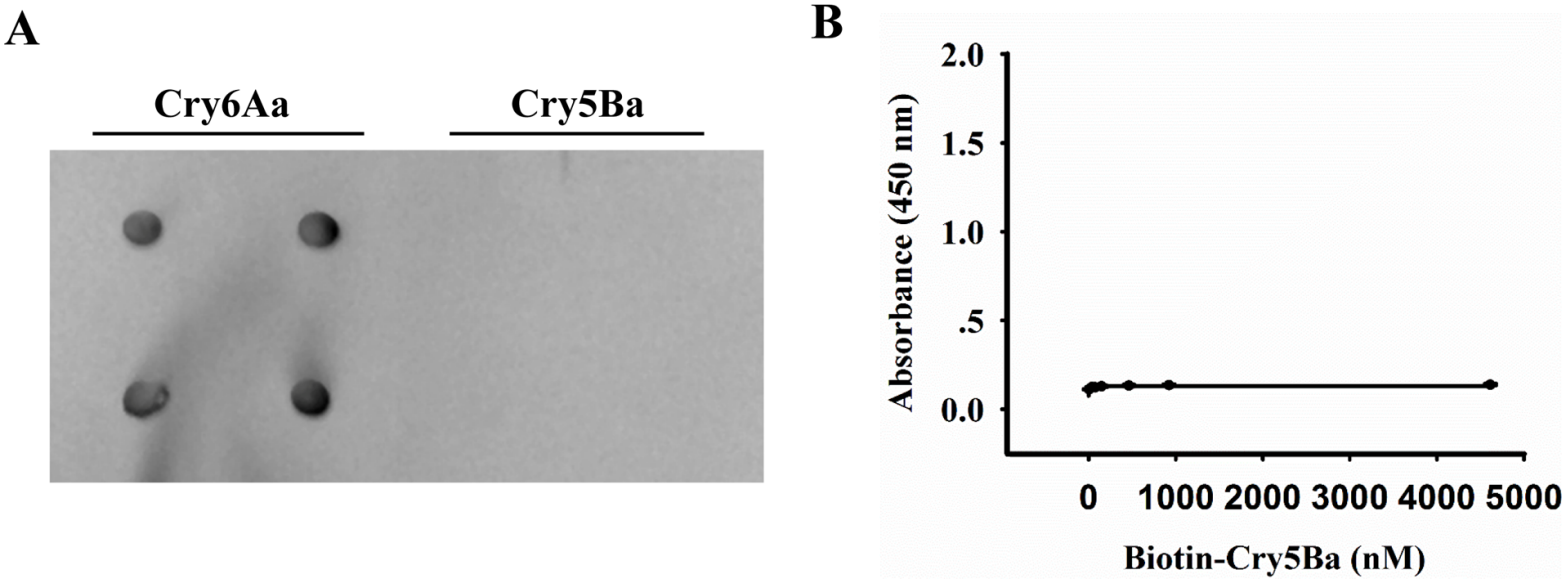
Cry5Ba is not able to bind to ASP-1. (A) Purified Cry5Ba and Cry6Aa were dotted on a NC membrane directly and were probed with biotin labeled ASP-1. Cry6Aa was the positive control. (B) Binding affinity of Cry5Ba to ASP-1 was determined by ELISA. Ninety-six-well microtiter plates coated with ASP-1 were incubated with increasing concentrations of biotinylated Cry5Ba.

To test whether Cry5Ba can bind to ASP-1, purified Cry5Ba and Cry6Aa was dotted on a membrane filter, and the filter was probed with biotin-labeled ASP-1. Cry6Aa was a positive control, and dot blot showed Cry5Ba did not bind to ASP-1 (Fig 10A). Ninety-six-well microtiter plates coated with ASP-1 were incubated with increasing concentrations of biotinylated Cry5Ba. ELISA showed Cry5Ba did not bind to ASP-1 (Fig 10B). These results indicate that Cry5Ba is not able to bind to ASP-1.

Calcium concentration can be assessed by measuring cytoplasmic fluorescence using the calcium indicator Fluo-4 AM [36]. Heat stroke was a positive control, as expected, heat stroke induced an increase in cytoplasm calcium concentration. However, killing with Cry5Ba did not affect intracellular calcium concentration (S8 Fig).

Together, these results indicate that the necrosis pathway is specifically induced by Cry6Aa, not by Cry5Ba.

## Discussion

The classical, pore-forming model is the widely accepted model for describing the mode of action of 3d-Cry. This model elucidates crystal protein action at the biochemical level, which includes crystal protein activation, receptor binding, pore formation, and cell lysis [10–13]. The identified receptors in the pore-forming model, such as cadherin, aminopeptidase (APN), and alkaline phosphatase (ALP), mediate insect resistance to 3d-Cry [45]. However, these receptors were not identified in *C*. *elegans* by the Cry6Aa-ligand blotting assay. These results indicate that the mode of action of non-3d crystal protein Cry6Aa against *C*. *elegans* may be different from the classical pore-forming model. The signal transduction model proposed that crystal proteins activate a Mg^2+^-dependent adenylyl cyclase (AC)/protein kinase A (PKA) signaling pathway [14,15]. This work show that crystal protein Cry6Aa triggers the Ca^2+^-dependent calpain–cathepsin necrosis pathway in *C*. *elegans*. Thus, in contrast to the classical pore-forming model, which elucidated at the biochemical level, the present findings reveal that crystal toxin triggers cell death by necrosis signaling pathway. This is the first demonstration that deficiency in necrosis pathway confers tolerance to Bt crystal protein.

Apoptosis occurs in embryonic and larval development. The CED-3 caspase, which is regulated by pro-apoptotic (CED-4, EGL-1) and anti-apoptotic (CED-9) factors, is involved in apoptosis [36,46]. However, we found that neither *ced-3 (n717)*, *ced-4 (n1162)*, nor *ced-9 (1950)* mutation changed Cry6Aa-induced death (S9 Fig). Therefore, cell death induced by Cry6Aa does not depend on the apoptotic machinery.

Autophagy is required for necrosis induced by prolonged hypoxia in *C*. *elegans* [47]. Conversely, autophagy protects *C*. *elegans* against necrosis during *Pseudomonas aeruginosa* infection [48]. *Lgg-1* encodes the *C*. *elegans* ortholog of Atg8/LC3 that facilitates autophagic vesicle growth, and *lgg-1* is involved for autophagy [38,49]. *Unc-51* encodes the serine threonine kinase ortholog of yeast autophagy protein Atg1 [50]. However, we found that neither *unc-51(e1189)* nor *lgg-1(bp500)* mutations suppressed or promoted Cry6Aa-induced death (S10 Fig). The above information suggest that autophagy plays different role respond to different pathological conditions.

An increasing number of bacterial pathogens have been shown to induce necrosis in host cells [2]. For example, *Erwinia carotovora*, *Photorhabdus luminescens*, and *Enterococcus faecalis* are reported to induce necrosis in *C*. *elegans* [51]. β-Toxin from *Clostridium perfringens*, alpha-toxin from *Clostridium septicum*, and *Helicobacter pylori* VacA [2] are reported to cause necrosis. Our study reveals that *B*. *thuringiensis* toxin triggers necrosis pathway in *C*. *elegans*. That Cry6A triggers necrosis represents a newly added necrosis paradigm in the *C*. *elegans*. Our findings contribute to the understanding of the mechanisms of host-pathogen interactions in higher species.

Necrosis plays an important role in host-pathogen interactions which can be used by both sides [2]. In some cases, necrosis plays a significant role in antiviral/antibacterial host defense [2,8]. For example, herpes simplex virus 1 (HSV-1) protein is reported to trigger an effective host-defense mechanism by activating RIP3/MLKL-dependent necrosis [52]. In others, necrosis does not as a host defense, but as pathogen survival strategy to aid its spread [2]. For example, cIAP2-dependent antagonism of RIPK3-mediated programmed necrosis critically protects the host from influenza infection [53]. Besides, it is reported that *E*. *carotovora* and *P*. *luminescens* used the necrosis to develop their effective virulence [51]. Our study reveals that deficiency in necrosis pathway confers tolerance to Bt crystal protein. It is possible that necrosis plays different role respond to different pathogens or toxins.

In the present work, evidence is presented that Bt crystal protein Cry6Aa triggers the necrosis pathway in *C*. *elegans* mediated by ASP-1, not by ASP-4 or ASP-3. To test whether ASP-1 is specifically required for Cry6A-induced necrosis, the *asp-1(tm666)* nematodes are exposed to two other death-inducing stimuli, heat stroke and hypoxia. We found that both mutant *asp-1(tm666)* and the wild-type N2 were sensitive to either heat stroke or hypoxia at the same level (S11 Fig). Thus, there is some difference in the necrosis pathway induced by Cry6Aa compared to other stressors, and ASP-1 is specifically required for Cry6A-induced necrosis. Previous studies of the cellular necrosis pathway have largely focused on neurodegeneration [43], where intrinsic insults induce necrosis mediated by ASP-3 and ASP-4 [44]. Recently, this pathway in the nematode intestine was reported, where lethal stress induced necrosis in *C*. *elegans* mediated by ASP-4 [43]. We recently found a two-domain protein named Nel, which is composed of a necrosis-inducing phytophthora protein 1-like domain found in phytopathogens and a ricin B-like lectin domain, induced necrosis mediated by ASP-4 [54]. Therefore, we speculate that different aspartyl proteases in the necrosis pathway respond to different death-inducing stimuli.

Our study show that Cry6A binds to ASP-1 and that ASP-1 mediates the protection and stabilization of Cry6Aa. This stabilization effect could be similar to that cadherin-mediated protection of Cry1Fa toxin from protease degradation in the insect gut [55]. ASP-1 is mainly distributed in the intestinal cells of *C*. *elegans* [28]. To explain how an intracellular ASP-1 protein binds Cry6Aa, we fed rhodamine labeled Cry6A to *C*. *elegans*, and monitored the signal of rhodamine-labeled crystal proteins with confocal microscope. As expected, the labeled Cry6A toxin was internalized into intestinal cells (S12 Fig).

A phylogenetic tree of the aspartyl proteases were constructed. ASP-1 from *C*. *elegans* belongs to the nematode-specific aspartyl protease class; however, ASP-3 and ASP-4 from *C*. *elegans* belong to a distinct branch that also includes mammalian proteases [44]. These results provide a plausible explanation for the lack of toxicity of Cry6Aa toward mammals. We hypothesize that the Bt non-three-domain Cry6Aa evolved to recognize the nematode specific ASP-1 and thus to target nematodes.

Griffitts *et al*. reported that *C*. *elegans* glycolipid mutants with resistance to Cry5Ba were not resistant to Cry6Aa [22]. In the present study, the *C*. *elegans* necrosis mutants with tolerant to Cry6Aa, were not tolerant to Cry5Ba. The above observations indicate that pathway of Cry6Aa against nematodes is different from that of Cry5Ba. A possible explanation for this difference is that the sequence and structure of the Cry6Aa toxin are different from those of Cry5Ba. The structure of Cry5Ba is similar to that of insecticidal 3d-Cry, and shows the typical conserved three-domain (3-d) architecture responsible for pore formation in insecticidal crystal proteins [20]. However, the amino acid sequence of Cry6Aa is completely different from that of 3d-Cry, and Cry6Aa does not show the typical 3-d architecture. Accordingly, further research is necessary to determine the structure of Cry6Aa.

Both *B*. *thuringiensis* and nematodes coexist in the soil ecosystem. We recently reported that nematodes are an alternative dominant host that contributes to the persistence, growth, and transmission of *B*. *thuringiensis* [56]. Some strains of *B*. *thuringiensis* have evolved to become nematode pathogens and use the host to reproduce, other non-nematicidal strains can reproduce on nematodes killed by other means or can still use nematodes as a means to reach their true host [56]. Bt has evolved crystal proteins to help it reproduction inside the host nematode [29]. On the other hand, *C*. *elegans* has evolved some conserved pathways to protect against *B*. *thuringiensis* crystal proteins [57,58]. Our study reveals that *B*. *thuringiensis* toxin triggers cell death by the necrosis pathway. Therefore, our findings have potential implications for understanding the co-evolution between nematodes and *B*. *thuringiensis*.

**Fig 11:**
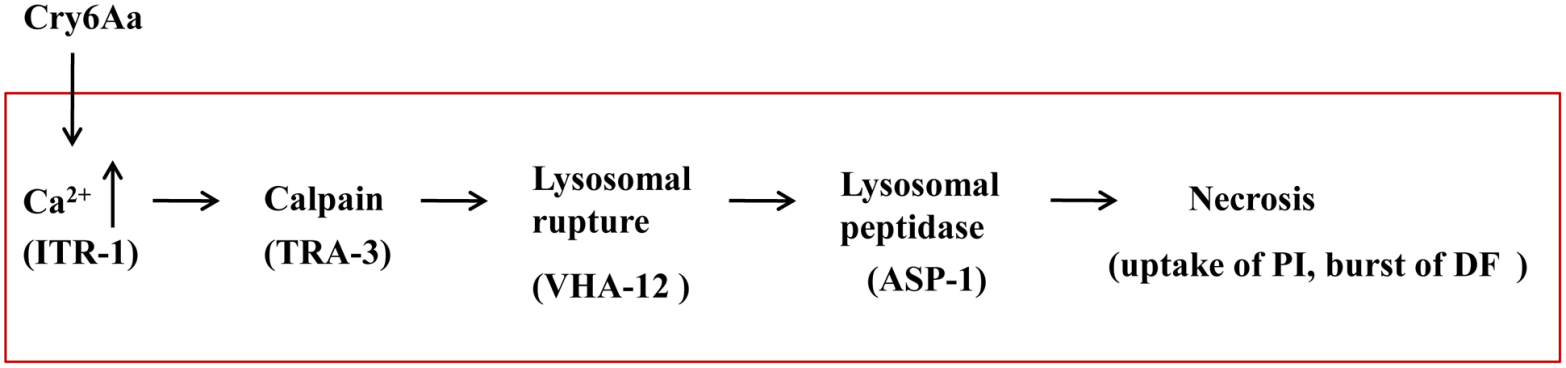
The working model of crystal protein Cry6Aa. Cry6Aa toxin triggers the Ca^2+^-dependent calpain–cathepsin necrosis pathway using Cry6Aa-*Caenorhabditis elegans* toxin-host interaction system, which involves an increase in concentrations of calcium, lysosomal lyses, the killer cathepsin proteases mediated by ASP-1, uptake of propidium iodide, and burst of death fluorescence. Abbreviations: PI, propidium iodide; DF, death fluorescence.

In conclusion, the present study reveals that crystal toxin triggers the necrosis pathway using Cry6Aa-*C*. *elegans* toxin-host interaction system, which involves an increase in concentrations of calcium, lysosomal lyses, uptake of propidium iodide, and burst of death fluorescence (Fig 11). We show that aspartic protease ASP-1 is required for Cry6Aa induced necrosis, whereas intrinsic insults induce necrosis mediated by ASP-3 and ASP-4. Understanding this model could lead to new strategies for nematode control. Further research is necessary to establish the existence of additional steps in Cry6Aa action: for example, what is the functional receptor in the intestinal surface and the role of receptor in crystal toxin action? Identification of the receptor would provide new avenues for studying the nematicidal mechanisms of Cry6Aa.

## Materials and Methods

### *C*. *elegans* culture and strains

Maintenance of *C*. *elegans* strains was performed as previously described [59]. The mutated and transgenic strains used in this study include the following (S4 Table): *ced-3(n717)*, *ced-4(n1162)*, *ced-9(n1950)*, *itr-1(sa73)*, *tra-3 (e1107)*, *vha-12(ok821)*, *asp-4(ok2693)*, *unc-51(e1189)*, *lgg-1(bp500)*, pwIs50[lmp-1::GFP + Cbr-unc-119(+)], and rnyEx109 [nhx-2p::D3cpv + pha-1(+)] were provided by the *Caenorhabditis* Genetics Center (http://www.cbs.umn.edu/CGC/). The *asp-3*(*tm4559*) and *asp-1* (*tm666*) (mutagen: TMP/UV) was obtained from Tokyo Women’s Medical College (http://www.wormbase.org/db/gene/variation?name=tm666;class=Variation). *C*. *elegans* was cultured using standard techniques, and the Bristol N2 strain was the wild type [59]. RnyEx109 is an integrated transgene that directs expression of calcium indicator d3cpv under the control of intestine-limited promoter Pnhx-2 [42,43]. PwIs50 is an integrated transgene with the intestinal lysosomal marker LMP-1:: GFP [39,40].

### Crystal protein purification and labeling

Cry6Aa and Cry5Ba proteins were purified according to our previously described method [60]. All purified protein samples were then solubilized in 20 mM HEPES (Calbiochem BB0364) (pH 8.0), quantified [61], and stored at -80°C. The purified Cry6Aa protein was labeled with N-hydroxysuccinimide-rhodamine (Pierce) or N-hydroxysulfosuccinimide ester-PC-biotin (Pierce), respectively, according to the manufacturer’s instructions.

### Nematode bioassays and lifespan analysis

The growth assay was performed according to the protocol described previously reported [62]. The LC_50_ assay was performed according to the protocol reported by Marroquin *et al* [22]. All lifespan measurements were performed on NGM agar plates of *E*. *coli* express Cry6Aa at 20°C [48]. The lifespan was monitored at 24 h, 48 h, 72 h, 96 h, 120 h, 144 h. Nematodes that did not move when they were gently prodded and displayed no pharyngeal pumping were marked as dead [48].

### Two-dimensional electrophoresis (2-DE)

Preparation of the total protein of *C*. *elegans* was conducted according to the protocol described by Schrimpf *et al* [63]. 2-DE was performed according to the protocol described by Krishnamoorthy *et al* [25]. Strips of 18 cm (pH 4–7) in length were used. After 2-DE, Gels were either silver stained and scanned using a GS-800 calibrated densitometer (Bio-Rad), or transferred to polyvinylidene difluoride Q (PVDF) membrane filters (Immobilon P, Millipore) for ligand blotting.

### Ligand blotting

Ligand blotting was performed according to the protocol reported by Fernandez-Luna *et al* [26]. Total *C*. *elegans* proteins were separated by 2-DE, and gels were transferred to a PVDF membrane in transfer buffer (20% methanol, 25 mM Tris-base, 192 mM glycine) for 60 min. Filters were blocked overnight in PBST (0.1% Tween 20 in phosphate buffered saline, pH 7.4) containing 3% BSA. Blocked filters were incubated with biotinylated Cry6Aa (10 nM) for 2 h at room temperature, and then washed three times using PBST. The bound protein was detected by 1 μg/ml streptavidin- horseradish peroxidase (HRP) conjugate (Sigma, S5512). The membrane was visualized using enhanced chemiluminescence substrate (SuperSignal West Pico, Pierce) following the manufacturer’s instructions.

### Western blotting

*C*. *elegans* treated with Cry6Aa were harvested and subsequently washed five times in M9 medium. Treated *C*. *elegans* samples were grinded with liquid nitrogen and were then subjected to sodium dodecyl sulfate polyacrylamide gel electrophoresis (SDS-PAGE) and transferred onto a PVDF membrane. Primary antibodies were anti-ASP-1 antibody (1:5000 dilution) for detection of ASP-1 expression. The secondary antibody was HRP-coupled anti-rabbit antibody (1:5000 dilution). The membrane was visualized as described above for the ligand blotting assay.

To monitor the size of Cry6Aa that is found inside the nematode, N2 and *asp-1(tm666)* were fed purified Cry6Aa proteins. Total proteins were then extracted from crystal protein treated nematodes, separated by SDS-PAGE, transferred onto a PVDF membrane. Primary antibodies were anti-Cry6Aa antibody (1:5000 dilution) for detection the size of Cry6Aa. The secondary antibody was HRP-coupled anti-rabbit antibody (1:5000 dilution). The membrane was visualized as described above for the ligand blotting assay.

### Dot blotting

Different quantities of ASP-1 were dotted onto a nitrocellulose (NC) membrane (Millipore, Bedford, MA, USA). After blocking with 3% bovine serum albumin (BSA) in PBST, NC membrane was bathed in biotinylated-Cry6Aa (10 nM) for 2 h at room temperature, washed three times using PBST. Unlabeled Cry6Aa (1000-fold excess) was used in the competition assays. The bound protein was detected by 1 μg/ml streptavidin-horseradish peroxidase (HRP) conjugate (Sigma, S5512). Finally, the signal was visualized using 3, 3'-diaminobenzidine tetrahydrochloride (DAB) substrate (Pierce) following the manufacturer’s instructions.

### Mass spectrometry

Selected spots were excised from the stained 2-DE gel and digested with trypsin, and the resulting peptide fragments were examined by mass spectroscopy according to the method described by Krishnamoorthy *et al* [25]. Peptides were examined using matrix-assisted laser desorption ionization-time of flight mass spectrometry (MALDI-TOF-MS) system (4700 Proteomics Analyzer, Applied Biosystems). A database search of spectral data was conducted using a mascot search engine (Matrix Science) to search the *C*. *elegans* databases.

### Gene cloning, expression, and purification of aspartic protease (ASP-1) in *E*. *coli*

RNA was extracted from *C*. *elegans* using a total RNA isolation system (Promega, Madison, WI, USA). First strand cDNA was synthesized from total RNA according to the manual (Takara, Tokyo, Japan). The ASP-1 full-length coding gene was amplified from synthesized cDNA by polymerase chain reaction (PCR) using a pair of primers based on the sequences reported for the aspartic protease gene (GenBank NM_171587): P1, 5′- CGCGAATTCATGCAGACCTTCGTTTT-3′, *Eco*RI restriction site is underlined; P2, 5′-CTGCTCGAGCTTACAATCCCTTGTGG-3′, *Xho*I restriction site is underlined. The amplified fragments were purified, digested with *Eco*RI and *Xho*I, and cloned into vector pGEX-6P-1 to create a recombinant vector pASP1-GST. Then, plasmid pASP1-GST was transformed into *E*. *coli* strain BL21 (DE3) (Amersham Biosciences, Uppsala, Sweden), and positive transformants were selected on an LB plate containing 100 μg/ml ampicillin. The ASP-1 was overexpressed in *E*. *coli* as a GST-tagged fusion protein ASP1-GST. ASP1-GST was purified according to the GST bind kit protocol.

### *Asp-1* rescue assays

pPD49.26 was used as the vector backbone for test constructs. The rescuing fragment comprising the entire 2715 bp *asp-1* gene (GenBank AF210248) contains 1421 bp 5′-flanking DNA of *asp-1*, 1191 bp *asp-1* cDNA, and 103 bp 3′-UTR of *asp-1*. The 2.7 kp rescuing fragment was made by PCR amplification with *Pfu* polymerase using a pair of primers designed based on the sequences reported for the aspartic protease gene: P3, 5′- CGCGAATTC CCAAAATGGGTCTTACC-3′, *Eco*RI restriction site is underlined; P4, 5′-CTGCTCGAG ATCAGAAATTAAAGATT-3′, *Xho*I restriction site is underlined. The amplified fragment was purified, digested with *Eco*RI and *Xho*I, and cloned into vector pPD49.26 to create a recombinant vector pASP1RES. This construct was co-injected at 10 ng/ml with the dominant rol-6 marker (pRF4) at 50 ng/ml into the gonads of nematode *asp-1* (*tm666*) by standard techniques [64]. The eggs of roller hermaphrodites were allowed to hatch to obtain L1 larvae, and then the L1 larvae were treated with Cry6Aa. The nematode resistance to Cry6Aa was evaluated based on the ability of L1 larvae to progress to adulthood during the course of the experiment [65].

### Enzyme-linked immunosorbent assay (ELISA)

The purified Cry6Aa was biotinylated using *N*-hydroxysulfosuccinimide ester-PC-biotin (Pierce, Rockford, IL) according to the manufacturer’s instructions. ELISA plates (high-binding, 96-well, Immulon 2HB; Thermo Fisher Scientific Inc., Waltham, MA) were incubated at 4°C for 12 h with 0.5 μg of ASP-1/well in 20 mM HEPES buffer (pH 8.0). The plates were then blocked at room temperature for 2 h in 100 μL PBST containing 3% BSA. For the binding assays, ELISA plates coated with ASP-1 were incubated with increasing concentrations of biotinylated Cry6Aa. For the competition assays, a 1000-fold molar excess of nonlabeled Cry6Aa was added to a solution that contained biotinylated Cry6Aa. Specific binding was determined by subtracting nonspecific binding (with 1000-fold molar excess of unlabeled Cry6Aa) from total binding (without excess unlabeled Cry6Aa). The other reaction conditions and the data analysis were conducted following the method described by Zhang et al [66]. Data were analyzed using SigmaPlot 12.0 software.

### Isothermal titration calorimetry (ITC)

Isothermal titration calorimetry experiments were performed at 25°C using the TAM Thermal Activity Monitor system (Thermometric AB, Sweden). Recombinant proteins ASP-1 were buffer-exchanged into 20 mM HEPES (pH 8.0) by G-25 spin-column chromatography. A solution containing 21.16 μM Cry6Aa in the same buffer was used as titrant, and solutions containing 2.20 μM ASP-1 were used in the calorimetry cell. The heat of reaction per injection was determined by integration of the peak areas using the Origin 8.6 software. ITC titration experiments were carried out with 20 injections, 10 μL per injection, and 75 s between each injection. Cry6Aa was titrated into 20 mM HEPES buffer to account for heat released due to dilution. Data were analyzed in Origin 8.6 software after subtracting the heat released from titrating Cry6Aa alone into buffer. The dissociation constants (Kd) were calculated from the plots of the total heat versus the molar ratio of Cry6Aa to ASP-1 [67].

### Proteolytic assays and protease protection assays

The experiments were based on the methodology of Fragoso *et al*. [68] with some modifications. Approximately 500 mg of N2 or *asp-1(tm666)* were triturated in 500 μL of acidic buffer (0.1 M sodium acetate and 0.5% v/v Triton-100; pH 4.8) at 4°C, centrifuged at 12,000g for 20 min, and the supernatant was used as crude protein extract. For proteolytic assays, 50 μL Cry6Aa was incubated with 50 μL crude protein extracts from wild type N2 or *asp-1(tm666)* for 2 h at 37°C. For protease protection assays, Cry6Aa was preincubated with ASP-1 or GST (control), and then treated with crude protein extracts from *asp-1(tm666)*. For the toxicity of the digested Cry6Aa, the sample buffer was replaced by HEPES buffer (pH 8.0), and the nematodes N2 were exposed to digested Cry6Aa for lifespan analysis.

### Death fluorescence (DF) measurements

Nematodes were imaged on a 2% agarose pad prepared on glass slides, and images were acquired using an Olympus BX63 microscope. Death fluorescence was observed through a DAPI filter (λ_ex_/λ_em_ 345 nm/455 nm). The fluorescence densities of nematodes were quantified using computerized image analysis with Olympus cellSens imaging software.

### Calcium imaging

*In vitro* changes in the concentrations of cytoplasmic calcium ([Ca^2+^]_i_) can be assessed by measuring the cytoplasmic fluorescence with the calcium indicator Fluo-4 AM (Molecular Probes) [36]. Nematodes were placed on a 2% agarose pad prepared on a glass slide, and then imaged under at 400 magnification on an Olympus BX63 microscope. The excitation and emission wavelengths were 489 nm and 508 nm, respectively. The relative fluorescence (F1/F0) was calculated using computerized image analysis with Olympus cellSens imaging software, where F1 was the fluorescence of *C*. *elegans* exposed to 63 μg/mL Cry6Aa protein for 6 days or heat stroke, and F0 was the fluorescence of *C*. *elegans* without Cry6Aa protein or heat stroke [36].

*In vivo* calcium levels were visualized using the calcium indicator d3cpv expressed from the intestine-limited promoter Pnhx-2 in transgenic (rnyEx109) nematodes [42,43]. Nematodes were imaged under Olympus FV1000 inverted confocal IX81 microscope. CFP (405 excitation, 480 emission), and FRET (405 excitation, 535 emission) filters and the FV10-ASW software were used to collect the FRET data. The FRET ratio was calculated by (FRET_int_−FRET_bkgnd_)/(CFP_int_−CFP_bkgnd_), where FRET_int_ and CFP_int_ represent the fluorescent intensities of the FRET and CFP channels of nematode gut, and FRET_bkgnd_ and CFP_bkgnd_ are the fluorescent intensities of FRET and CFP in the background region [41,42].

### Lysosome-like gut granule visualization

*In vitro* lysosomal rupture can be assessed by labeling with lysotracker [43] (Life Technologies, USA). The excitation and emission wavelengths were 555 nm and 580 nm, respectively. *In vivo*, a *C*. *elegans* transgenic (pwIs50) strain expressing the intestinal lysosomal marker LMP-1::GFP [39,40] was used to examine lysosome integrity. Nematodes were placed on a 2% agarose pad prepared on a glass slide, and then imaged under 400 magnification on an Olympus BX63 microscope. The excitation and emission wavelengths were 489 nm and 508 nm, respectively.

### Nematode heat stroke, hypoxia and propidium iodide staining assays

Nematode heat stroke, hypoxia and propidium iodide staining assays were performed according to the protocol reported by Nikos Kourtis *et al* [35]. Pepstatin A (an aspartyl proteases inhibitor) and Z-Val-Phe-CHO (a calpain inhibitor) were used as necrosis inhibitors [44] in this study. After propidium iodide (Sigma) staining, worms were visualized using an Olympus BX63 microscope. The excitation and emission wavelengths were 555 nm and 580 nm, respectively.

### Statistics

The results presented in each figure are the average of three independent experiments. LC_50_ values were calculated using PROBIT analysis [69]. The three independent LC_50_ were averaged and showed as mean ± SD. The significance of the differences between two datasets was assessed by Student’s *t* test.

## Supporting Information

S1 Fig Detection and identification of Cry6Aa binding proteins from *C*. *elegans* using 2-D electrophoresis and ligand blotting. For 2-DE, *C*. *elegans* proteins were resolved by isoelectric focusing using pH 4–7, 18 cm strips followed by separation on an SDS-PAGE gel. Gels were either silver stained (A) or transferred to PVDF filters and probed with biotin-Cry6Aa (B). The positions of molecular size markers (kDa) are indicated on the side of the gel. Arrows denote positions of the major Cry6Aa binding proteins mentioned in the results.
(TIF)

S2 Fig Detection of Cry6Aa after ingestion by nematode N2 and *asp-1(tm666)* by Western blot analysis. N2 and *asp-1(tm666)* were incubated with Cry6Aa protein, and then detected by Western blot using an anti-Cry6Aa antibody. Line 1, Controls of crystal protein Cry6Aa without being incubated by nematode. Line 2, Cry6Aa incubated by wild type N2. Line 3, Cry6Aa incubated by *asp-1(tm666)*.
(TIF)

S3 Fig ASP-1-mediated protection of Cry6Aa in *C*. *elegans*. (A) The proteolytic digestion of Cry6Aa protoxin (lane 1) by crude protein extracts from wild type nematodes N2 (lane 2), mutant *asp-1(tm666)* (lane 3), Cry6Aa was incubated with ASP-1 (lane 4) or GST (lane 5), and then exposed to extracts from *asp-1(tm666)*. (B) Survival of N2 exposed to Cry6Aa proteolytic digestion in crude protein extracts from N2, mutant *asp-1(tm666)* in the presence or absence of ASP-1. Data were showed as mean ± SD (n = 3).
(TIF)

S4 Fig The uptake of propidium iodide was significantly suppressed in necrosis mutants *itr-1(sa73)* (A), *tra-3(e1107)* (B), and *vha-12(ok821)* (C) after exposure to Cry6Aa. These images should be compared to the necrosis induced in wild type N2 from Fig 3. Fluorescence microscopy was used to monitor propidium iodide uptake. One of the fluorescent images were magnified (boxed inset). The numbers of cell corpses per nematode were counted (Right). These results are the mean ± SD of three independent experiments. Double asterisks indicate p < 0.01. The bar denotes 20 μm.
(TIF)

S5 Fig Intestinal cell plasma membrane integrity was lost in *asp-3(tm4559)* (A), and *asp-4(ok2693)* (B) after exposure to Cry6Aa. These images should be compared to the necrosis induced in wildtype N2 from Fig 3. Fluorescence microscopy was used to monitor propidium iodide uptake. Arrows indicate intestinal cells stained with propidium iodide due to loss of membrane integrity. One of the fluorescent images were magnified (boxed inset). The numbers of cell corpses per nematode were counted (Right). These results are the mean ± SD of three independent experiments. The bar denotes 20 μm.
(TIF)

S6 Fig Heat stroke results in a burst of death fluorescence in *C*. *elegans*. DIC and the fluorescence microscopy of N2 with or without heat stroke. Typical blue fluorescence increased in N2 after exposure to heat stroke. One of three representative experiments is shown. The bar denotes 20 μm.
(TIF)

S7 Fig ASP-1 is required for the Cry6Aa-induced necrosis pathway. (A) The effects of ASP-1 on the Cry6Aa-induced burst of death fluorescence. Typical fluorescence increased in N2 but not in *asp-1(tm666)* after exposure to Cry6Aa. The right part shows the quantification of the death fluorescence levels in N2 and *asp-1(tm666)* after exposure to Cry6Aa. These results are the mean ± SD of three independent experiments. Double asterisks indicate p < 0.01. The bar denotes 20 μm. (B) The uptake of propidium iodide was significantly suppressed in *asp-1(tm666)* after exposure to Cry6Aa. These images should be compared to the necrosis induced in wild type N2 from Fig 3. Fluorescence microscopy was used to monitor propidium iodide uptake. The numbers of cell corpses per nematode were counted (Right). These results are the mean ± SD of three independent experiments. Double asterisks indicate p < 0.01. The bar denotes 20 μm. (C) The effects of ASP-1 on Cry6Aa-induced intestinal cell lysosomal rupture. DIC and the fluorescence microscopy of *asp-1(tm666)* labeled with the intestinal lysosomal marker lysotracker.
(TIF)

S8 Fig Cry5Ba did not induce an increase in cytoplasm calcium concentration in *C*. *elegans*. Fluorescence microscopy was used to monitor calcium concentration by measuring cytoplasmic fluorescence using the calcium indicator Fluo-4 AM. The right part shows the quantification of the fluorescence levels. Heat stroke was a positive control. These results are the mean ± SD of three independent experiments. A single asterisk indicates p < 0.05. The bar denotes 20 μm.
(TIF)

S9 Fig The susceptibility of apoptosis mutants to Cry6Aa. Survival of *ced-3(n717)*, *ced-4(n1162)*, and *ced-9(n1950*) apoptosis mutants exposed to Cry6Aa. Data were showed as mean ± SD (n = 3).
(TIF)

S10 Fig The susceptibility of autophagy mutants to Cry6Aa. Survival of *unc-51(e1189)* and *lgg-1(bp500)* autophagy mutants exposed to Cry6Aa. Data were showed as mean ± SD (n = 3).
(TIF)

S11 Fig The susceptibility of *asp-1 (tm666)* to two other death-inducing stimuli, heat stroke and hypoxia. Survival of *asp-1 (tm666)* exposed to heat stroke and hypoxia. Data were showed as mean ± SD (n = 3).
(TIF)

S12 Fig Confocal laser scanning microscope image showed uptake of Cry6Aa toxin into nematode gut cells. Nematode were fed rhodamine-labeled Cry6Aa toxins, and then imaged using the rhodamine channel to visualize toxin (left panels), the bright-field to visualize the nematode (middle panels) and merged image (right panels). Toxin was detected inside the nematode gut cells, but not in the control (CK). One of three representative experiments is shown. The bar denotes 21.53 μm.
(TIF)

S1 Table PMF results and database searches using the Mascot program and NCBInr database (DOC)

S2 Table Data analysis of growth assay for *asp-1(tm666)* response to Cry6Aa (DOC)

S3 Table Data analysis of mortality assay for *asp-1(tm666)*, *itr-1(sa73)*, *tra-3(e1107)*, *vha-12(ok821)*, *asp-3* (*tm4559*) and *asp-4* (*ok2693*) response to Cry6Aa. (DOC)

S4 Table The information of gene and protein mentioned in this study (DOC)
