# Supplementary figures and images for: Bacillus thuringiensis Crystal Protein Cry6Aa Triggers Caenorhabditis elegans Necrosis Pathway Mediated by Aspartic Protease (ASP-1)
Source: PLoS Pathog. 2016 Jan 21;12(1):e1005389. doi: 10.1371/journal.ppat.1005389 (PMC4721865; doi:10.1371/journal.ppat.1005389)

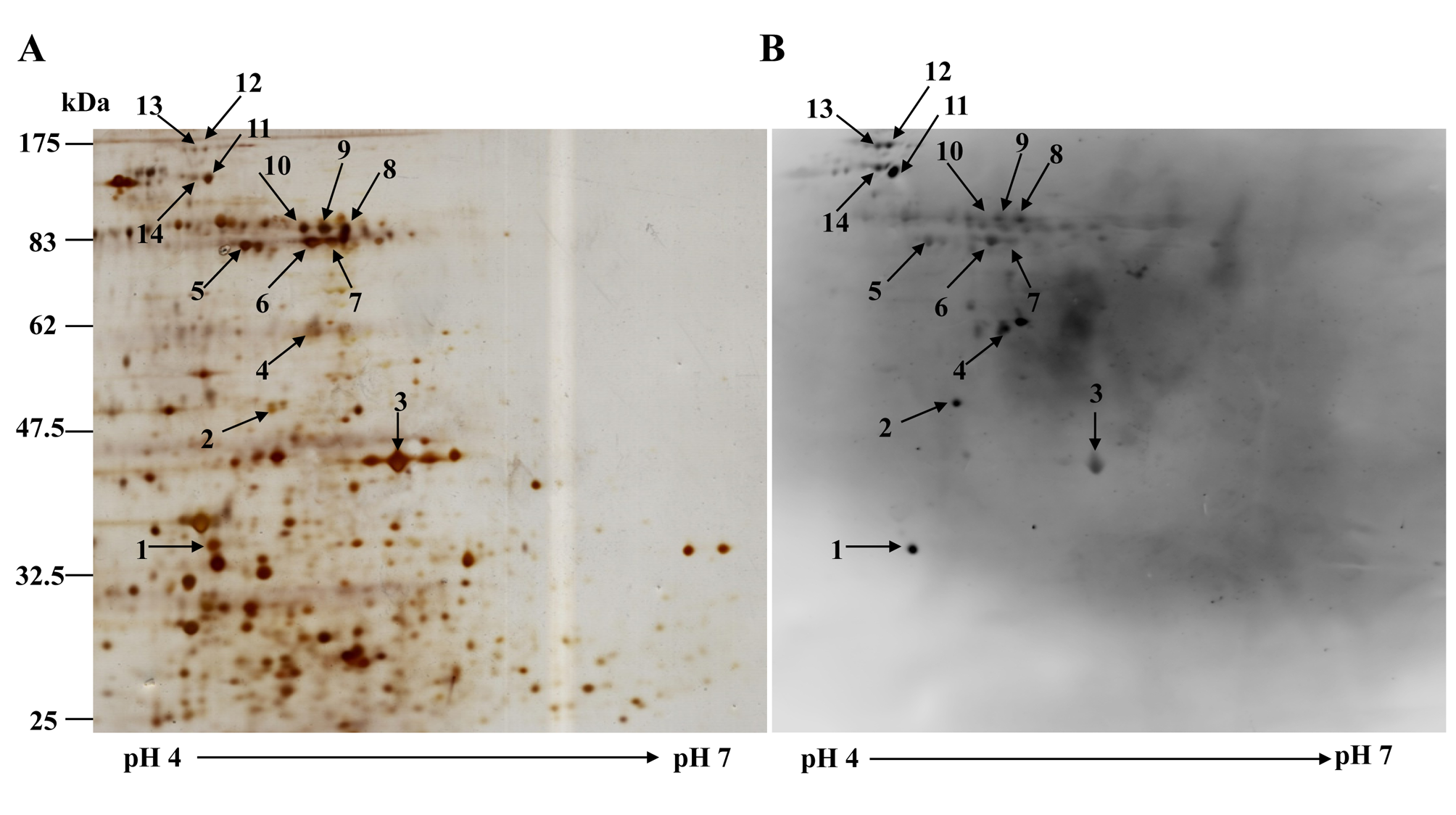

Supplement: S1 Fig — For 2-DE, C. elegans proteins were resolved by isoelectric focusing using pH 4–7, 18 cm strips followed by separation on an SDS-PAGE gel. Gels were either silver stained (A) or transferred to PVDF filters and probed with biotin-Cry6Aa (B). The positions of molecular size markers (kDa) are indicated on the side of the gel. Arrows denote positions of the major Cry6Aa binding proteins mentioned in the results. (TIF) [file ppat.1005389.s001.tif]

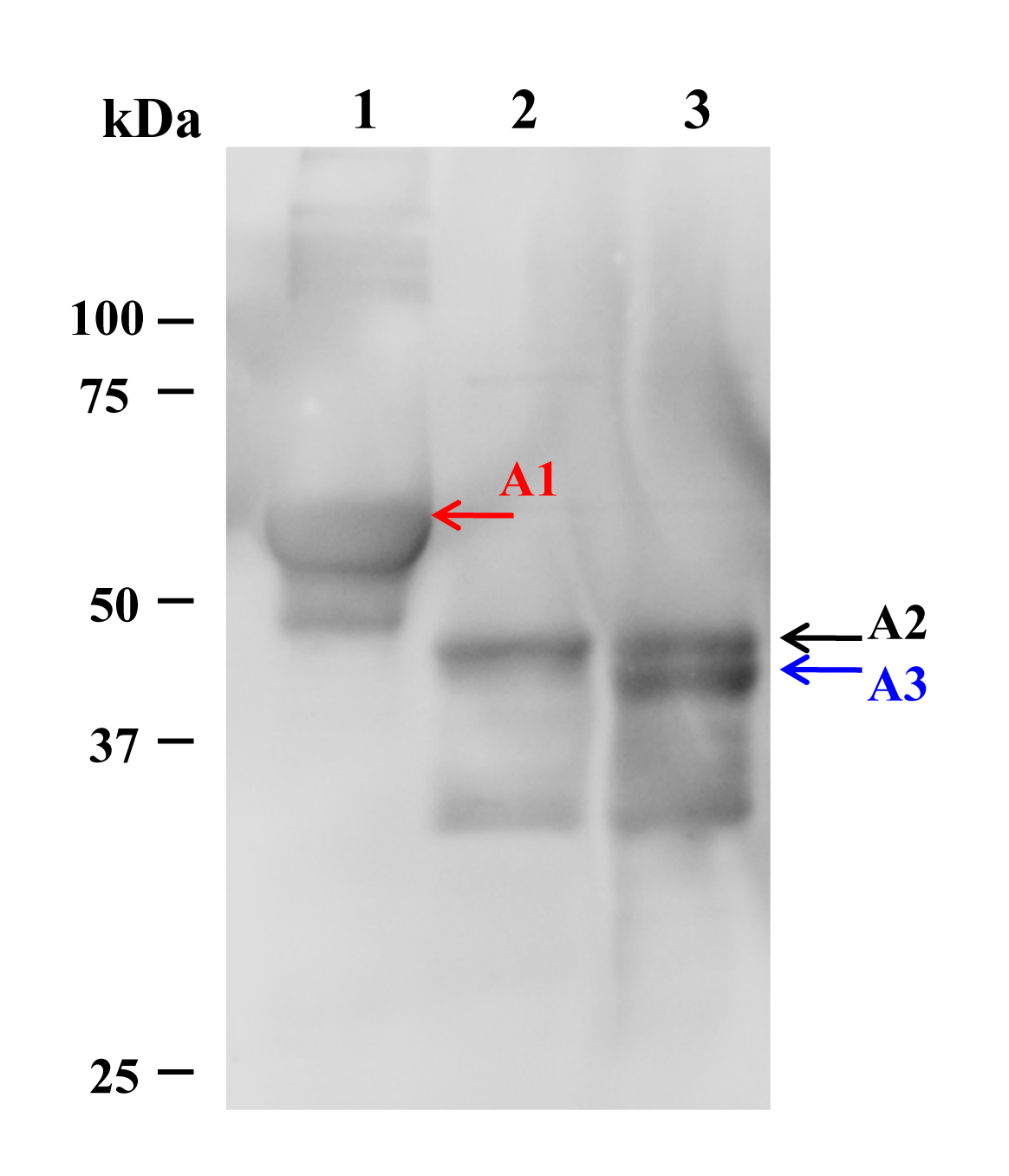

Supplement: S2 Fig — N2 and asp-1(tm666) were incubated with Cry6Aa protein, and then detected by Western blot using an anti-Cry6Aa antibody. Line 1, Controls of crystal protein Cry6Aa without being incubated by nematode. Line 2, Cry6Aa incubated by wild type N2. Line 3, Cry6Aa incubated by asp-1(tm666). (TIF) [file ppat.1005389.s002.tif]

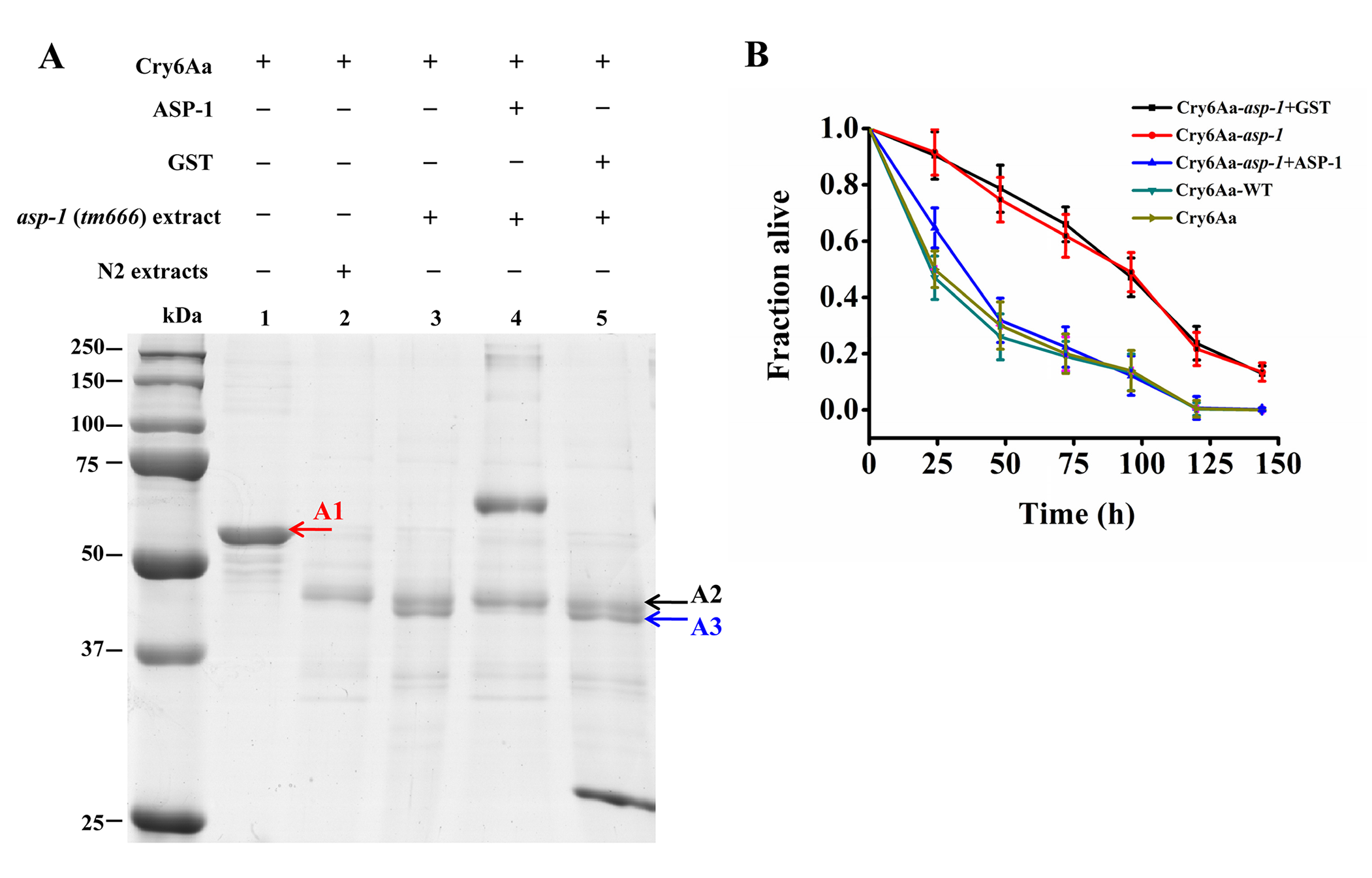

Supplement: S3 Fig — (A) The proteolytic digestion of Cry6Aa protoxin (lane 1) by crude protein extracts from wild type nematodes N2 (lane 2), mutant asp-1(tm666) (lane 3), Cry6Aa was incubated with ASP-1 (lane 4) or GST (lane 5), and then exposed to extracts from asp-1(tm666). (B) Survival of N2 exposed to Cry6Aa proteolytic digestion in crude protein extracts from N2, mutant asp-1(tm666) in the presence or absence of ASP-1. Data were showed as mean ± SD (n = 3). (TIF) [file ppat.1005389.s003.tif]

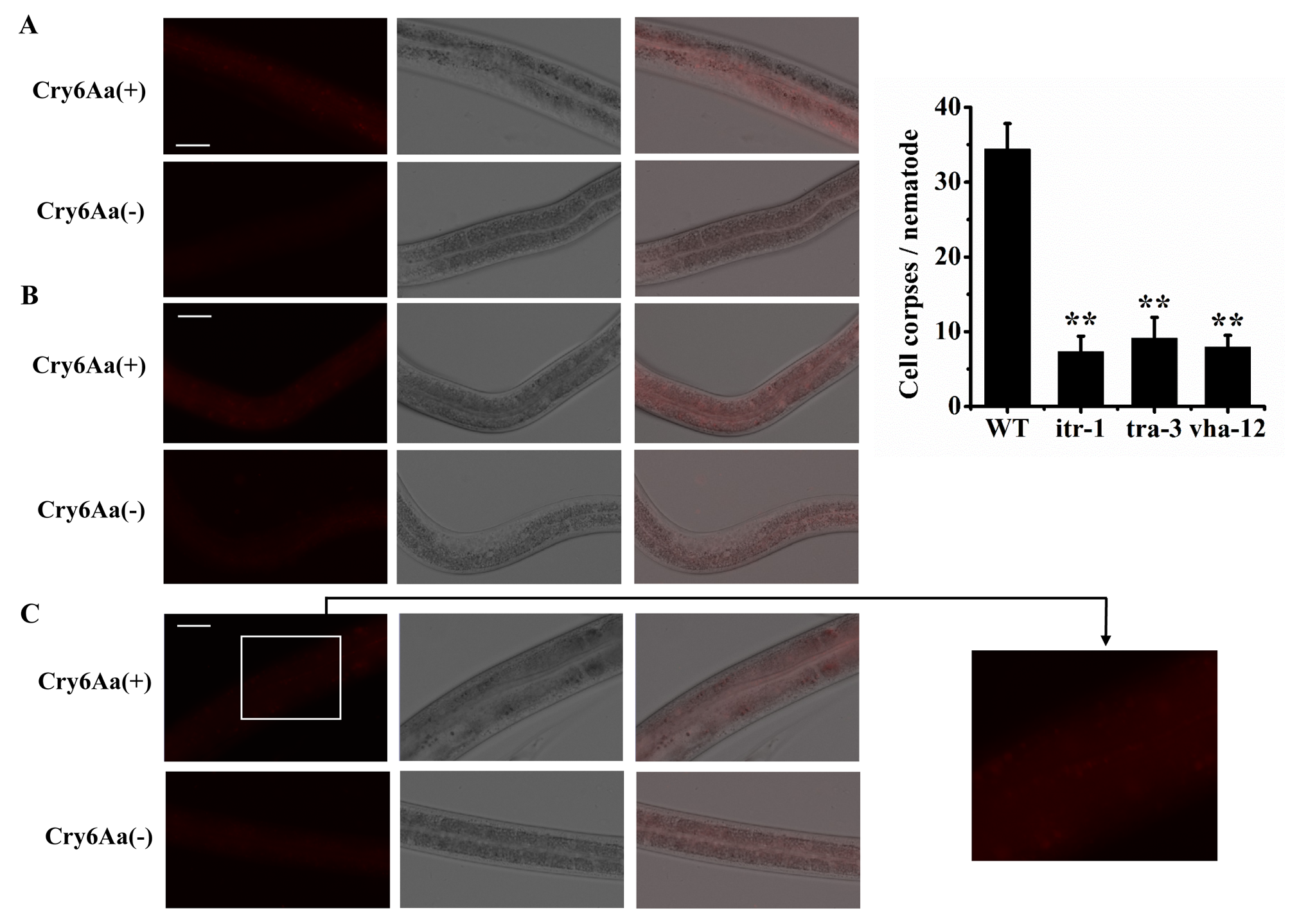

Supplement: S4 Fig — These images should be compared to the necrosis induced in wild type N2 from Fig 3. Fluorescence microscopy was used to monitor propidium iodide uptake. One of the fluorescent images were magnified (boxed inset). The numbers of cell corpses per nematode were counted (Right). These results are the mean ± SD of three independent experiments. Double asterisks indicate p < 0.01. The bar denotes 20 μm. (TIF) [file ppat.1005389.s004.tif]

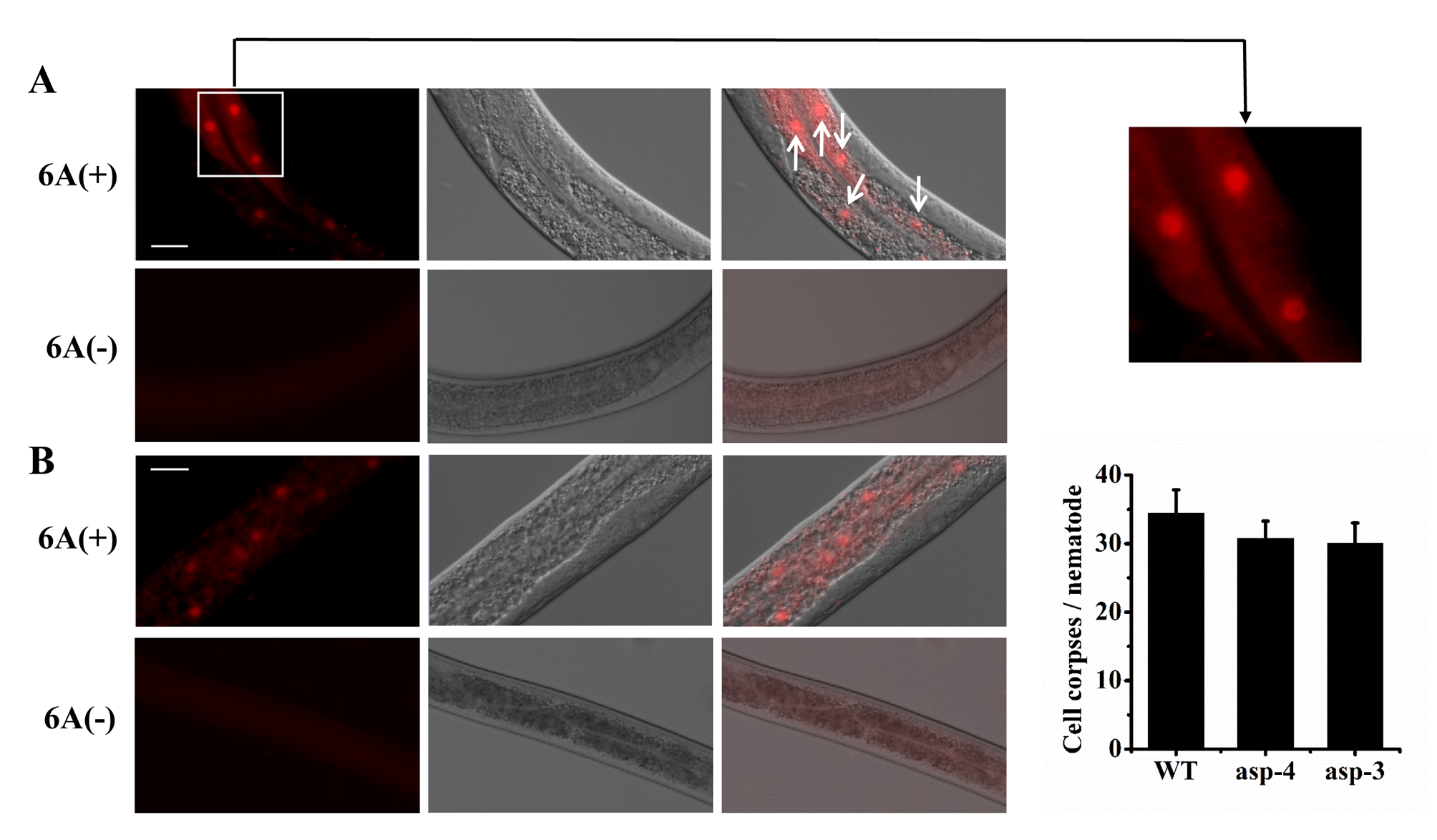

Supplement: S5 Fig — These images should be compared to the necrosis induced in wildtype N2 from Fig 3. Fluorescence microscopy was used to monitor propidium iodide uptake. Arrows indicate intestinal cells stained with propidium iodide due to loss of membrane integrity. One of the fluorescent images were magnified (boxed inset). The numbers of cell corpses per nematode were counted (Right). These results are the mean ± SD of three independent experiments. The bar denotes 20 μm. (TIF) [file ppat.1005389.s005.tif]

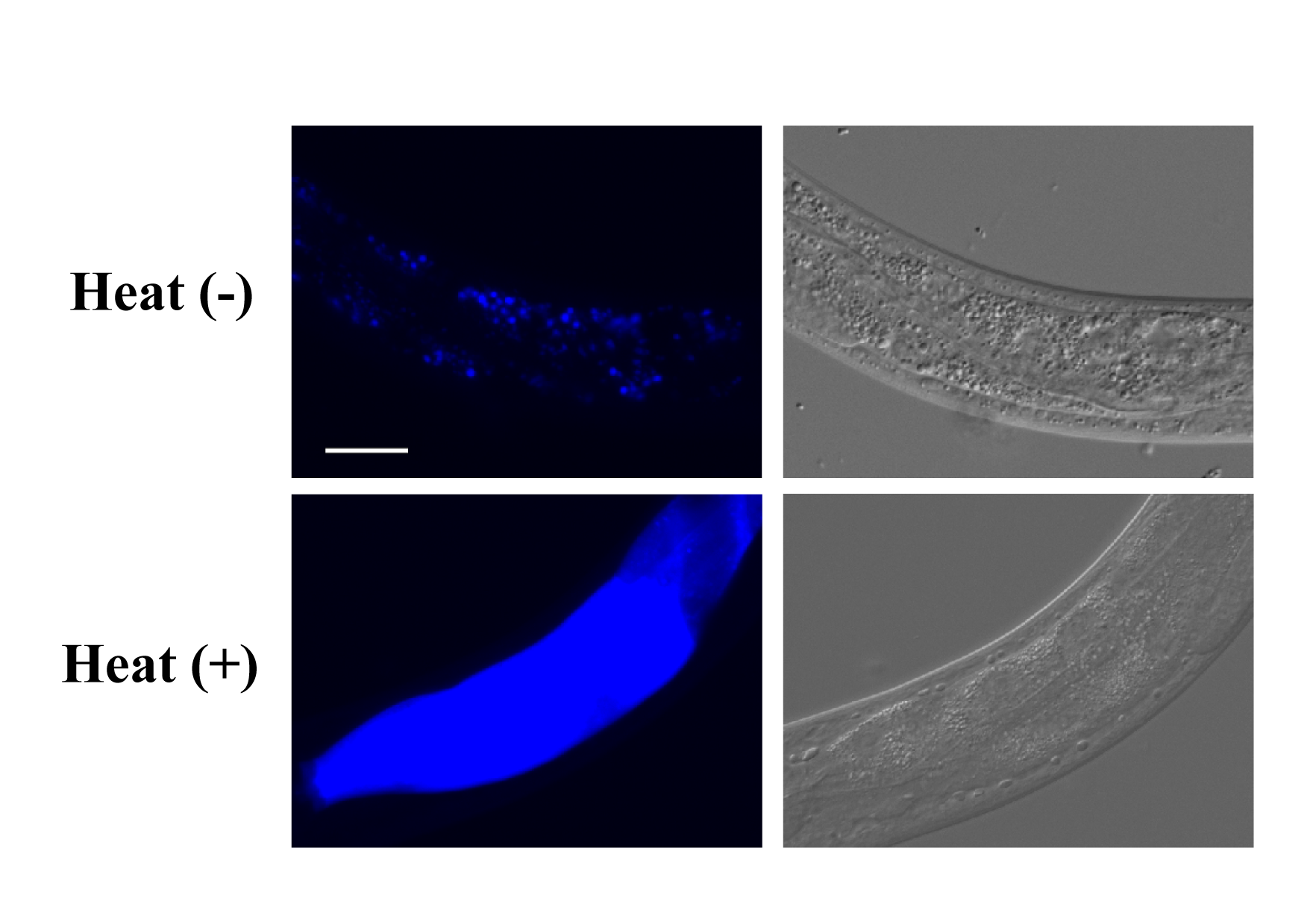

Supplement: S6 Fig — DIC and the fluorescence microscopy of N2 with or without heat stroke. Typical blue fluorescence increased in N2 after exposure to heat stroke. One of three representative experiments is shown. The bar denotes 20 μm. (TIF) [file ppat.1005389.s006.tif]

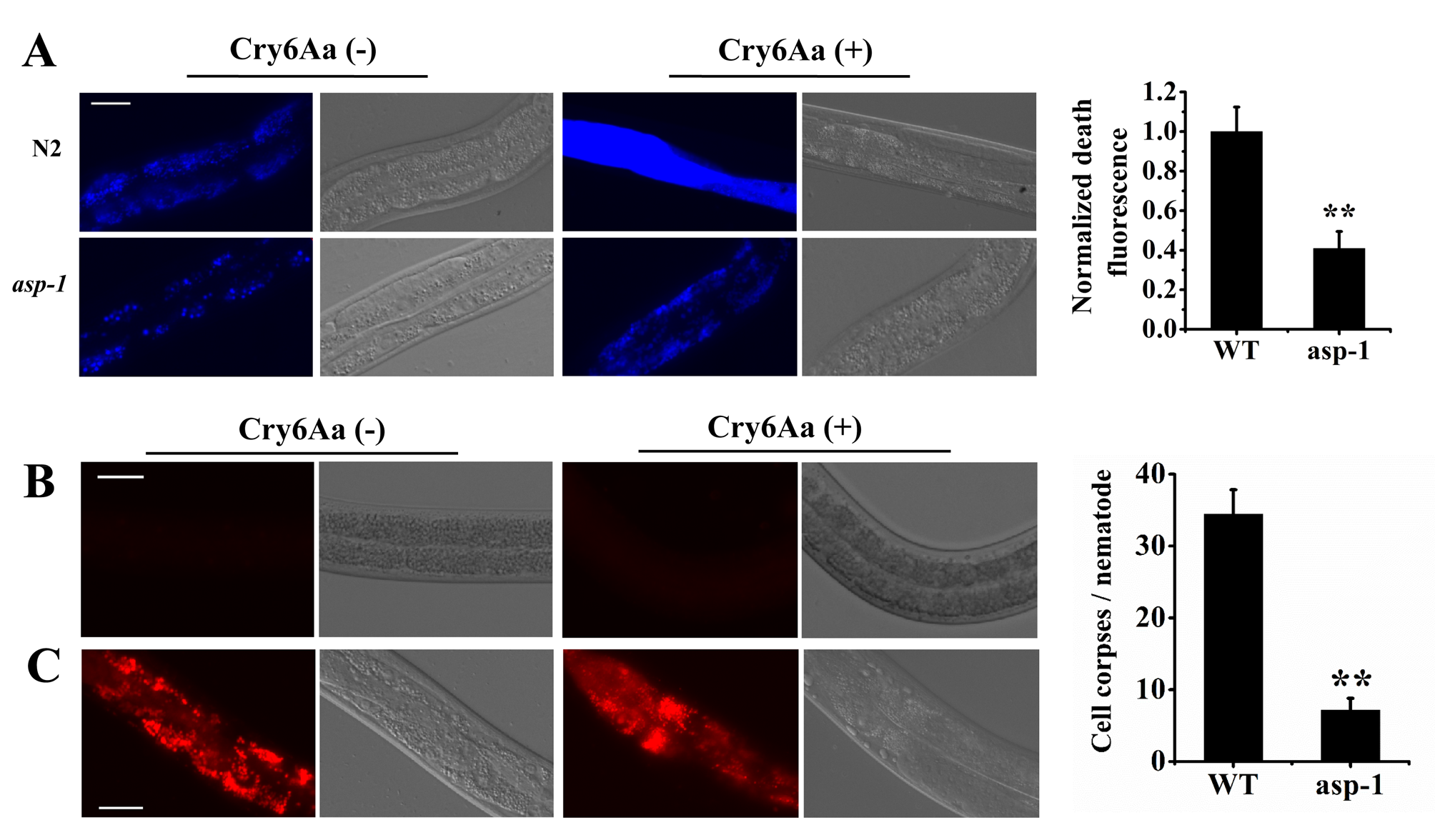

Supplement: S7 Fig — (A) The effects of ASP-1 on the Cry6Aa-induced burst of death fluorescence. Typical fluorescence increased in N2 but not in asp-1(tm666) after exposure to Cry6Aa. The right part shows the quantification of the death fluorescence levels in N2 and asp-1(tm666) after exposure to Cry6Aa. These results are the mean ± SD of three independent experiments. Double asterisks indicate p < 0.01. The bar denotes 20 μm. (B) The uptake of propidium iodide was significantly suppressed in asp-1(tm666) after exposure to Cry6Aa. These images should be compared to the necrosis induced in wild type N2 from Fig 3. Fluorescence microscopy was used to monitor propidium iodide uptake. The numbers of cell corpses per nematode were counted (Right). These results are the mean ± SD of three independent experiments. Double asterisks indicate p < 0.01. The bar denotes 20 μm. (C) The effects of ASP-1 on Cry6Aa-induced intestinal cell lysosomal rupture. DIC and the fluorescence microscopy of asp-1(tm666) labeled with the intestinal lysosomal marker lysotracker. (TIF) [file ppat.1005389.s007.tif]

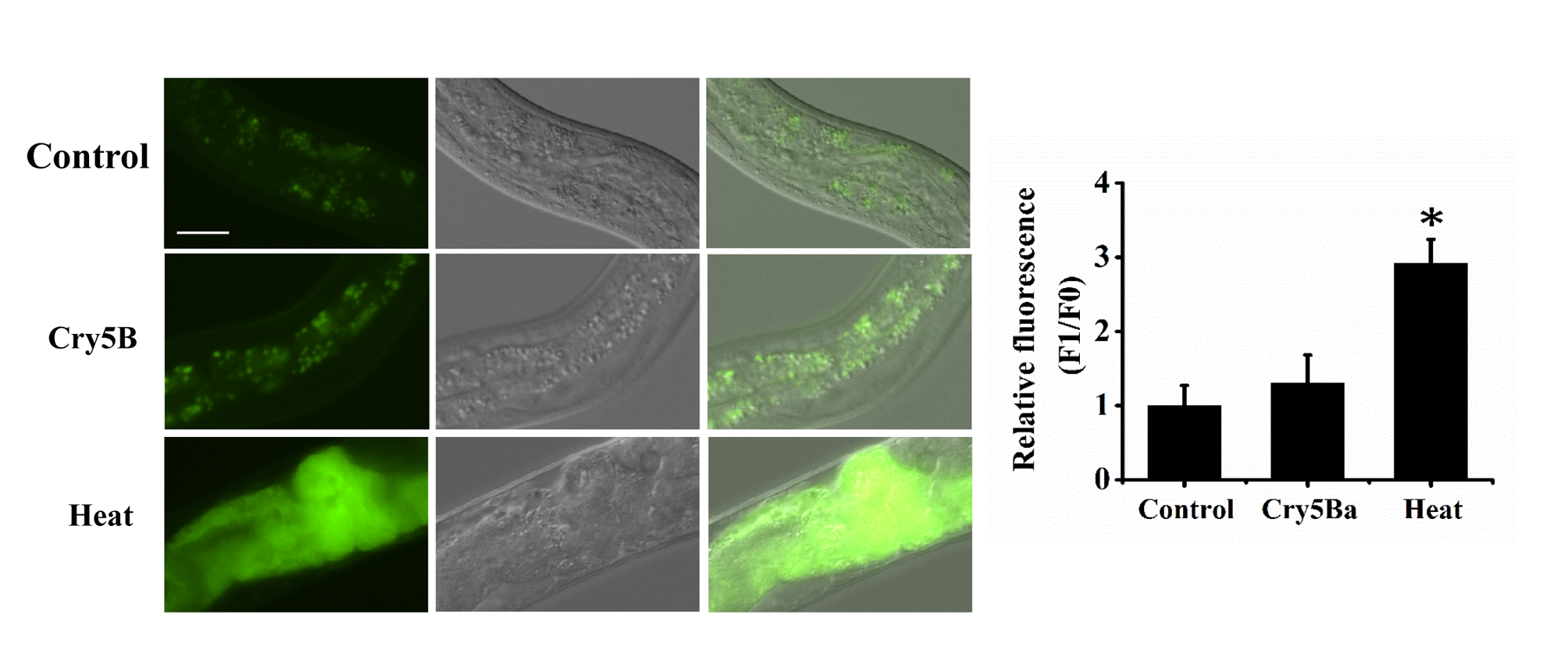

Supplement: S8 Fig — Fluorescence microscopy was used to monitor calcium concentration by measuring cytoplasmic fluorescence using the calcium indicator Fluo-4 AM. The right part shows the quantification of the fluorescence levels. Heat stroke was a positive control. These results are the mean ± SD of three independent experiments. A single asterisk indicates p < 0.05. The bar denotes 20 μm. (TIF) [file ppat.1005389.s008.tif]

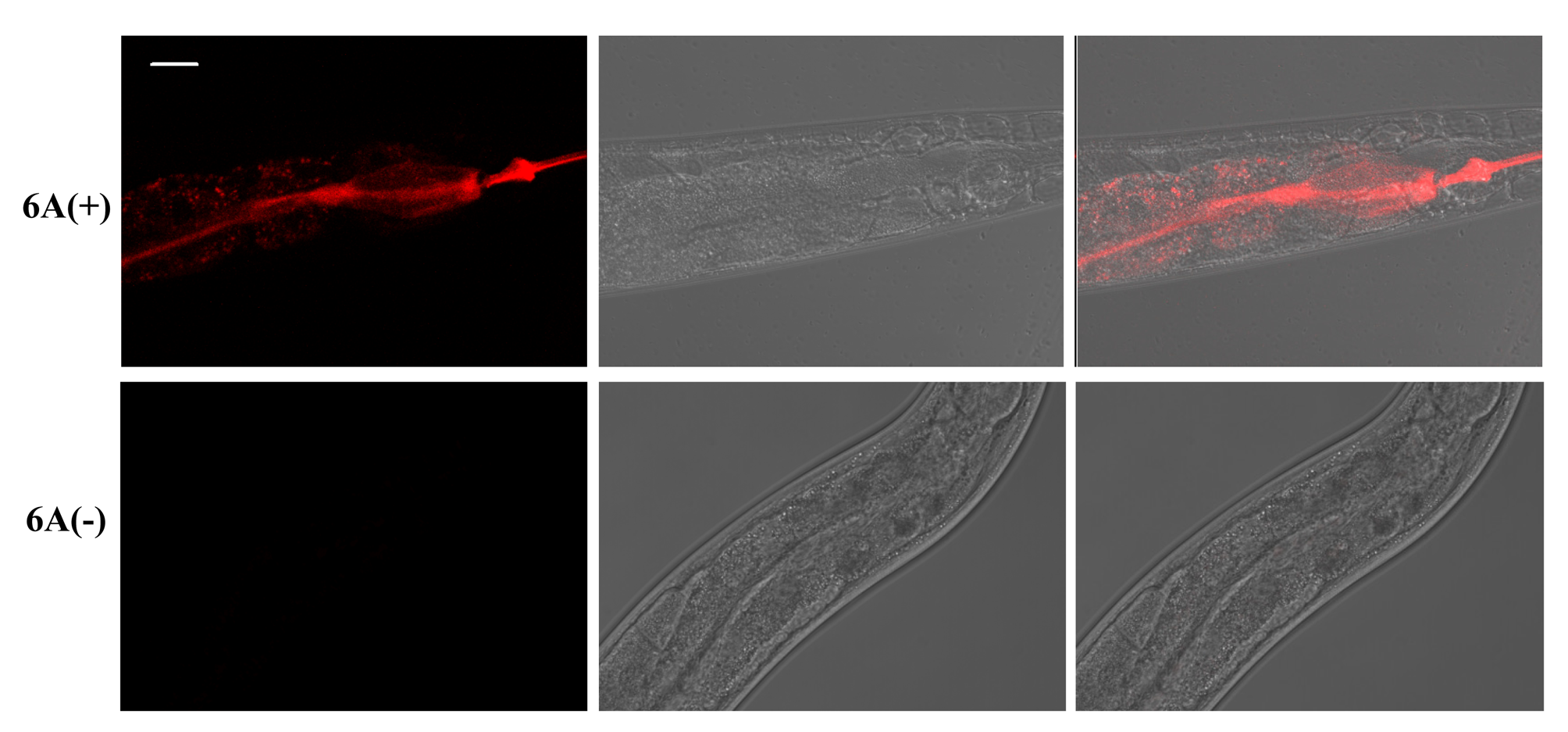

Supplement: S12 Fig — Nematode were fed rhodamine-labeled Cry6Aa toxins, and then imaged using the rhodamine channel to visualize toxin (left panels), the bright-field to visualize the nematode (middle panels) and merged image (right panels). Toxin was detected inside the nematode gut cells, but not in the control (CK). One of three representative experiments is shown. The bar denotes 21.53 μm. (TIF) [file ppat.1005389.s012.tif]
