# Supplementary material for: Bacillus thuringiensis Crystal Protein Cry6Aa Triggers Caenorhabditis elegans Necrosis Pathway Mediated by Aspartic Protease (ASP-1)
Source: PLoS Pathog. 2016 Jan 21;12(1):e1005389. doi: 10.1371/journal.ppat.1005389 (PMC4721865; doi:10.1371/journal.ppat.1005389)
Supplement: S1 Table — (DOC) [file ppat.1005389.s013.doc]

**Table S**1. PMF results and database searches using the Mascot program and NCBInr database

| Spots | Proteins | NCBI accession No. | | Mascot  Score^a^ | Analysis | Mr^b^ (kDa) | pI^c^ | Sequence coverage^d^ (%) | Species |
| --- | --- | --- | --- | --- | --- | --- | --- | --- | --- |
| **Physiology and Metabolism related proteins** | | | | | | | | | |
| 1 | ASP-1 | NP_741677 | | 70 | MS (PMF) | 43.12 | 5.81 | 38 | *C. elegans* |
| 2 | ATP-2 | NP_498111 | | 69 | MS (PMF) | 57.66 | 5.52 | 31 | *C. elegans* |
| **Cytoskeletal proteins** | | | | | | | | | |
| 3 | Actin | 1D4X_A | | 185 | MS (PMF) | 41.98 | 5.30 | 59 | *C. elegans* |
|  | Actin-3 | NP_505817 | | 185 | MS (PMF) | 42.11 | 5.30 | 59 | *C. elegans* |
|  | Actin-4 | NP_001024803 | | 167 | MS (PMF) | 40.63 | 5.56 | 53 | *C. elegans* |
|  | Actin | CAA34719 | | 157 | MS (PMF) | 42.02 | 5.30 | 55 | *C. elegans* |
|  | Actin-2 | NP_505818 | | 145 | MS (PMF) | 42.09 | 5.29 | 43 | *C. elegans* |
| **Unknown** | | | | | | | | | |
| 4 | F58E2.4 | NP_500352 | 61 | | MS (PMF) | 80.06 | 7.61 | 19 | *C. elegans* |

^a^ The Mascot Score is given as S=–10*log (P), where P is the probability that the observed match is a random event. It is an indication of match quality. ^b^ Predicted mass (Mr). ^c^ Predicted isoelectric point (pI). ^d^ Sequence coverage (%) is defined as the ratio of the length of the query sequence covered by matched peptides to the whole protein sequence. Abbreviations: ASP-1, Aspartic proteases; ATP-2, ATP synthase subunit family member; *C. elegans*, *Caenorhabditis elegans*
