# Supplementary material for: Bacillus thuringiensis Crystal Protein Cry6Aa Triggers Caenorhabditis elegans Necrosis Pathway Mediated by Aspartic Protease (ASP-1)
Source: PLoS Pathog. 2016 Jan 21;12(1):e1005389. doi: 10.1371/journal.ppat.1005389 (PMC4721865; doi:10.1371/journal.ppat.1005389)
Supplement: S2 Table — (DOC) [file ppat.1005389.s014.doc]

**Table S2. Data analysis of growth assay for *asp-1(tm666)* response to Cry6Aa**

| Strains | GI_50_ (μg/ml) | Standard  deviation | p value relative to N2 | | Relative sensitivity GI_50_ mutant /GI_50_ N2 |
| --- | --- | --- | --- | --- | --- |
| N2 | 4.9 | 0.6 | |  |  |
| *asp-1(tm666)* | 45.1 | 3.7 | | <0.01 | 9.2 |
