# Supplementary material for: Bacillus thuringiensis Crystal Protein Cry6Aa Triggers Caenorhabditis elegans Necrosis Pathway Mediated by Aspartic Protease (ASP-1)
Source: PLoS Pathog. 2016 Jan 21;12(1):e1005389. doi: 10.1371/journal.ppat.1005389 (PMC4721865; doi:10.1371/journal.ppat.1005389)
Supplement: S3 Table — (DOC) [file ppat.1005389.s015.doc]

**Table S3. Data analysis of mortality assay for *itr-1(sa73)*, *tra-3(e1107)*, *vha-12(ok821)*, *asp-3* (*tm4559*) and *asp-4* (*ok2693*) response to Cry6Aa**

| Strains | LC_50_ (μg/ml) | Standard  deviation | p value relative to N2 | Relative sensitivity LC_50_ mutant /LC_50_ N2 |
| --- | --- | --- | --- | --- |
| N2 | 63.7 | 6.8 |  |  |
| *asp-1(tm666)* | 715.6 | 69.3 | <0.01 | 11.23 |
| *itr-1(sa73)* | 614.9 | 70.6 | <0.01 | 9.65 |
| *tra-3(e1107)* | 539.8 | 81.4 | <0.01 | 8.47 |
| *vha-12(ok821)* | 594.2 | 68.5 | <0.01 | 9.33 |
| *asp-3(tm4559)* | 79.1 | 9.7 | >0.05 | 1.24 |
| *asp-4(ok2693)* | 73.5 | 9.2 | >0.05 | 1.15 |
