# Supplementary material for: Bacillus thuringiensis Crystal Protein Cry6Aa Triggers Caenorhabditis elegans Necrosis Pathway Mediated by Aspartic Protease (ASP-1)
Source: PLoS Pathog. 2016 Jan 21;12(1):e1005389. doi: 10.1371/journal.ppat.1005389 (PMC4721865; doi:10.1371/journal.ppat.1005389)
Supplement: S4 Table — (DOC) [file ppat.1005389.s016.doc]

**Table S4. The information of gene and protein mentioned in this study**

| Gene No. | Gene | | Gene description | Species |
| --- | --- | --- | --- | --- |
| 21311782 | *cry6Aa* | | Nematicidal crystal protein | *B. thuringiensis* |
| 159137702 | *cry5Ba* | | Nematicidal crystal protein | *B. thuringiensis* |
| [180251](http://www.ncbi.nlm.nih.gov/sites/entrez?db=gene&cmd=Retrieve&dopt=full_report&list_uids=180251) | | *asp-1* | Aspartyl protease | *C. elegans* |
| [180947](http://www.ncbi.nlm.nih.gov/sites/entrez?db=gene&cmd=Retrieve&dopt=full_report&list_uids=180947) | | *asp-3* | Aspartyl protease | *C. elegans* |
| [181444](http://www.ncbi.nlm.nih.gov/sites/entrez?db=gene&cmd=Retrieve&dopt=full_report&list_uids=181444) | | *asp-4* | Aspartyl protease | *C. elegans* |
| [177546](http://www.ncbi.nlm.nih.gov/sites/entrez?db=gene&cmd=Retrieve&dopt=full_report&list_uids=177546) | | *itr-1* | Inositol triphosphate receptor | *C. elegans* |
| 178385 | | *tra-3* | Calpain protease | *C. elegans* |
| [180692](http://www.ncbi.nlm.nih.gov/sites/entrez?db=gene&cmd=Retrieve&dopt=full_report&list_uids=180692) | | *vha-12* | Vacuolar H^+^-ATPase B subunit | *C. elegans* |
| [178272](http://www.ncbi.nlm.nih.gov/sites/entrez?db=gene&cmd=Retrieve&dopt=full_report&list_uids=178272) | | *ced-3* | Cell death protein | *C. elegans* |
| [175643](http://www.ncbi.nlm.nih.gov/sites/entrez?db=gene&cmd=Retrieve&dopt=full_report&list_uids=175643) | | *ced-4* | Cell death protein | *C. elegans* |
| [3565776](http://www.ncbi.nlm.nih.gov/sites/entrez?db=gene&cmd=Retrieve&dopt=full_report&list_uids=3565776) | | *ced-9* | Apoptosis regulator | *C. elegans* |
| [180311](http://www.ncbi.nlm.nih.gov/sites/entrez?db=gene&cmd=Retrieve&dopt=full_report&list_uids=180311) | | *unc-51* | serine threonine kinase | *C. elegans* |
| [174050](http://www.ncbi.nlm.nih.gov/sites/entrez?db=gene&cmd=Retrieve&dopt=full_report&list_uids=174050) | | *lgg-1* | [Autophagosomal protein](http://www.tandfonline.com/doi/abs/10.4161/auto.6.5.12252) | *C. elegans* |
